# Patient-reported outcome measures for assessing health-related quality of life in people with type 2 diabetes: A systematic review

**DOI:** 10.1007/s11154-022-09734-9

**Published:** 2022-07-02

**Authors:** Marlous Langendoen-Gort, Lenka Groeneveld, Cecilia A. C. Prinsen, Joline W. Beulens, Petra J. M. Elders, Ilana Halperin, Geetha Mukerji, Caroline B. Terwee, Femke Rutters

**Affiliations:** 1 General Practice, Amsterdam UMC, Location Vrije Universiteit Amsterdam, de Boelelaan 1117, Amsterdam, Netherlands; 2 Epidemiology and Data Science, Amsterdam UMC, Location Vrije Universiteit Amsterdam, de Boelelaan 1117, Amsterdam, Netherlands; 3 Amsterdam Public Health, Health Behaviors & Chronic Diseases, Amsterdam, The Netherlands; 4 Amsterdam Public Health, Methodology, Amsterdam, The Netherlands; 5 Amsterdam Cardiovascular Sciences, Diabetes & Metabolism, Amsterdam, The Netherlands; 6 Department of Medicine, Temerty Faculty of Medicine, Sunnybrook Health Sciences Center, King’s College Circle, University of Toronto, Toronto, ON Canada; 7 Women’s College Hospital Institute for Health System Solutions and Virtual Care, 76 Grenville Street, Toronto, ON Canada

**Keywords:** Systematic review, Type 2 diabetes, Patient-reported outcome measures, Health-related quality of life

## Abstract

Patient-Reported Outcome Measures (PROMs) are important tools to assess outcomes relevant to patients, with Health-Related Quality Of Life (HRQOL) as an important construct to be measured. Many different HRQOL PROMs are used in the type 2 diabetes field, however a complete overview of these PROMs is currently lacking. We therefore aimed to systematically describe and classify the content of all PROMs that have specifically been developed or validated to measure (aspects of) HRQOL in people with type 2 diabetes. A literature search was performed in PubMed and EMBASE until 31 December 2021. Studies on the development or validation of a PROM measuring HRQOL, or aspects of HRQOL, in people with type 2 diabetes were included. Title and abstract and full-text screening were conducted by two independent researchers and data extraction was performed independently by one of the researchers. Data were extracted on language in which the PROM was developed, target population, construct(s) being measured, names of (sub)scales and number of items per (sub)scale. In addition, all PROMs and subscales were classified according to specific aspects of HRQOL based on the Wilson & Cleary model (symptom status, functional status, general health perceptions) to aid researchers in PROM selection. In total 220 studies were identified that developed or validated PROMs that measure (aspects of) HRQOL in people with type 2 diabetes. Of the 116 unique HRQOL PROMs, 91 (of the subscales) measured symptom status, 60 measured functional status and 26 measured general health perceptions. In addition, 16 of the PROMs (subscales) measured global quality of life. 61 of the 116 PROMs (subscales) also include characteristics of the individual (e.g. aspects of personality, coping) or environment (e.g. social or financial support) and patient-reported experience measures (PREMs, e.g. measure of a patient's perception of their personal experience of the healthcare they have received, e.g. treatment satisfaction), which are not part of the HRQOL construct. Only 9 of the 116 PROMs measure all aspects of HRQOL based on the Wilson & Cleary model. Finally, 8 of the 116 PROMs stating to measure HRQOL, measured no HRQOL construct. In conclusion, a large number of PROMs are available for people with type 2 diabetes, which intend to measure (aspects of) HRQOL. These PROMs measure a large variety of (sub)constructs, which are not all HRQOL constructs, with a small amount of PROMs not measuring HRQOL at all. There is a need for consensus on which aspects of HRQOL should be measured in people with type 2 diabetes and which PROMs to use in research and daily practice. PROSPERO: CRD42017071012. COMET database: http://www.comet-initiative.org/studies/details/956.

**Supplementary information:**

The online version contains supplementary material available at 10.1007/s11154-022-09734-9.

## Introduction

Due to the high global prevalence of type 2 diabetes (˜400 million) combined with the chronic nature of the disease, it is important to measure outcomes that matter most to patients [1, 2]. This can be done by measuring patient-reported outcomes (PROs). PROs are health outcomes directly reported by patients about how they feel or function in relation to a health condition. In clinical research and care an important PRO to measure is (aspects of) health-related quality of life (HRQOL), including symptom status, functional status and general health perceptions [3]. The terms HRQOL and Quality of Life (QOL) are often used interchangeably. However many authors state that (overall) QOL is a broader concept, referring to how happy or satisfied a person is with his/her life as a whole [4–6]. Clinicians and researchers in the medical field generally prefer to measure only those aspects of QOL related to health (often referred to as HRQOL) instead of QOL, because the non-medical aspects of QOL are outside the scope of health care interventions. Not only in care, but also clinical trials, the measurement of HRQOL is becoming increasingly important.

One of the most often used conceptual models of HRQOL was developed by Wilson and Cleary [4]. The model contains five levels of outcomes, namely biological and psychological variables, symptom status (including disease specific symptoms, physical symptoms and mental symptoms), functional status (including physical function, psychological function and social/role function), general health perceptions and overall quality of life (including overall quality of life, well-being and life satisfaction). In this review, we define HRQOL as symptoms, functional status and general health perceptions.

To date, many different PRO measurement instruments (PROMs) are available that measure HRQOL in people with type 2 diabetes, identified by previous reviews [7–16]. However, these reviews included studies in both people with type 1 and 2 diabetes, which represent different pathologies and large differences in age, and therefore different PROs may be relevant or the validity and reliability of PROMs may be different in people with type 1 versus type 2 diabetes [7, 8, 11]. Other reviews only included patients with amputations [14], only PROMs measuring one aspect of HRQOL, e.g. depressive symptoms [11] or were conducted over 10 years ago [9, 12]. A recent review by Wee et al. 2021 aimed to identify all PROMs used for people with diabetes [15]. However, Wee et al. did not classify (subscales of) PROMs according to which specific aspects of HRQOL, based on the Wilson & Cleary model, they measure. This classification is important because instrument selection should be based on the relevant aspects of HRQOL to measure, not on available PROMs, which are mostly multi-dimensional instruments that measure many different things. Therefore, the content and quality of PROMs should be evaluated for each PROM separately. Furthermore, often questionnaires that are being referred to as HRQOL PROMs include (subscales) that measure non-HRQOL aspects, such as characteristics of the individual, overall quality of life, or even patient-reported experience measures (PREMs), which are not part of the HRQOL construct according to the Wilson and Cleary model. This has not been made clear in previous reviews. Because of these research gaps, we aimed to systematically describe and classify the content of all PROMs that have specifically been developed or validated to measure (aspects of) HRQOL in people with type 2 diabetes.

## Methods

This systematic review has been conducted in accordance with the Preferred Reporting Items for Systematic Reviews and Meta-Analysis (PRISMA) statement [17] and the COSMIN guideline for conducting systematic reviews [18]. The protocol was registered in the PROSPERO database on 2 July 2017 (registration number CRD42017071012).

### Literature search

The databases PubMed and EMBASE were searched from date of inception until May 2019 and then updated until 31^st^ of December 2021. This literature search has been performed by researcher CBT in cooperation with a medical librarian from the Amsterdam UMC, Amsterdam, the Netherlands. The search strategy was built up around three blocks of search terms, namely type 2 diabetes, measurement properties (i.e. different search terms for reliability, validity, responsiveness and interpretability) and PROMs (i.e. different search terms for report, questionnaire and survey). For the type 2 diabetes dimension search terms were used to identify studies that focused on people with type 2 diabetes. For finding studies on measurement properties a highly sensitive validated search filter was used [19] and a comprehensive PROM filter, developed by the Patient Reported Outcomes Measurement Group, University of Oxford and available through the COSMIN website, was used to search for PROMs [20]. An overview of the search strategy can be found in Appendix 1.

All identified studies were uploaded in Covidence [21], which is an online platform that supports researchers in conducting systematic reviews by enabling them to upload all of the identified studies, screening of the studies on title and abstract and full-text, resolve disagreements, and export data. Covidence was used during the study to remove duplicates and for the screening and selection process of the retrieved studies.

## Study selection

Pairs of two researchers (JWB, PJME, AAH, IH, MLG, GM, CACP, FR, CBT and MW) independently reviewed the identified studies based on title and abstract and full-text article. In case of any disagreements between two of the researchers a third researcher was consulted to reach consensus. From the identified studies reference lists of the included articles were checked by one of the researchers (MLG or FR) to search for additional eligible studies, after which pairs of researchers reviewed the studies found through reference search. The screening and selection process was conducted based on pre-defined eligibility criteria.

A study was included when it met all five of the following inclusion criteria: (I) the authors aimed to develop a PROM, evaluate the measurement properties or evaluate the interpretability (e.g. floor and ceiling effects) of a PROM, (II) it concerned a PROM that aims (according to the authors of the included papers) to measure at least (aspects of) symptom status, functional status, general health perceptions or HRQOL based on the model of Wilson and Cleary [4], (III) the PROM is filled in by the patient in self-report, interview or diary form or is completed on behalf of the patient (proxy), (IV) > 50% of the study population consisted of people with type 2 diabetes, as reported in the article or when it could be assumed based on age and type of diabetes medication, or studies that reported measurement properties specifically for a subgroup of people with type 2 diabetes, and (V) the article is available in full-text. There were no restrictions on language in which the article was written.

A study was excluded when any of the following exclusion criteria were met: the PROM (I) was only used as a determinant or outcome measure or was used as a comparison instrument in a validation study of another instrument, (II) solely measured characteristics of the individual or behaviors (e.g. aspects of personality, self-efficacy, coping and eating behavior), characteristics of the environment (e.g. social support and financial support), patient-reported experience measures (PREM, i.e. a measure of a patient's perception of their personal experience of the healthcare they have received, e.g. treatment satisfaction) or overall quality of life (QOL) (e.g. well-being or satisfaction with life in general), or (III) was primarily developed for screening, diagnostic or prognostic purposes. PROMs that measure a combination of (aspects of) HRQOL as well as other constructs were included if the main aim was to measure (aspects of) HRQQL.

### Data synthesis

Information from the included studies was systematically synthesized by one of the researchers (LG, MLG or FR). In case of any uncertainties a second researcher (CBT) was consulted. The characteristics of the PROM, including official name, language in which the PROM was developed, target population for which the PROM was developed (including type 1 or type 2 diabetes), construct(s) being measured, name of (sub)scales as well as number of items were extracted using a study-specific and pilot-tested PROM characteristics table. If necessary, relevant comments were also recorded. With regard to the (sub)scales, we extracted the number of items per subscale and the original names when possible. However, some studies did not clearly mention the number of items per subscale or the names of the subscales and then we noted the total number of items and for the names we either used a name that matched the authors’ description of the subscales or when the authors added or eliminated only a few items (not changing the scales), we used the subscale name of the original instrument.

All PROM (subscales) were classified according to the constructs of HRQOL measured, based on the Wilson & Cleary model [4]. This classification was based on reviewing the names of the (sub)scales and not the content of the PROMs. Some (sub)scales did not measure aspects of HRQOL, but were classified as measures of overall quality of life (including well-being and life satisfaction), characteristics of the individual/environment or PREM. If information on PROM characteristics could not be found in the paper, additional resources such as other articles, Google (e.g. manuals or websites) or the PROQOLID database [22] were consulted.

## Results

**Fig. 1:**
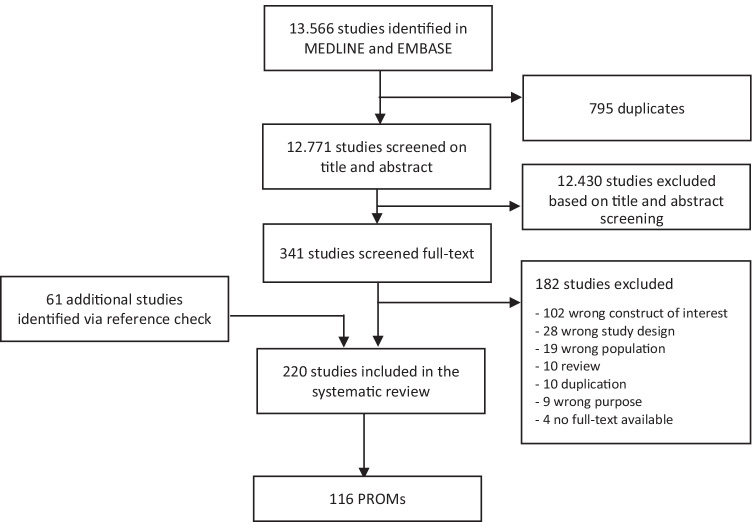
Flowchart of the screening and selection process

Figure 1 represents the flowchart of the screening and selection process.

### Characteristics of the PROMs

A total of 116 unique HRQOL PROMs were identified, of which 82 (70.7%) were specifically developed for people with (type 1 and 2) diabetes (Table 1). Other PROMs were validated in people with type 2 diabetes, but were originally developed for 21 different target populations, the main one being the general population, namely 20/116 (17.2%). The PROMs were developed in 32 different languages, most often in English (N = 68), Dutch (N = 9), Japanese (N = 7) and Spanish (N = 7). 7/116 (6.0%) PROM were developed in more than one language at the same time, such as the World Health Organisation Quality of Life (WHOQOL-100) [23] and the World Health Organisation Quality of Life (WHOQOL)-BREF [24, 25]. For all 116 PROMs, the number of (sub-)scales varied from 1 to 21.

**Table 1:** Characteristics of the included HRQOL PROMs

**Reference**	**PROMs**	**Development language**	**Development target population**	**Construct(s) being measured**	**Names of (sub)scales**	**Number of items per (sub)scale**	**Comments**
Akinci et al. [57]	15D standardized measure of health-related quality of life Finnish (15D Finnish)	Finnish	General population	Generic health-related quality of life	1. Breathing 2. Mental function 3. Speech 4. Vision 5. Mobility 6. Usual activities 7. Vitality 8. Hearing 9. Eating 10. Elimination 11. Sleeping 12. Distress 13. Discomfort and its symptoms 14. Sexual activity 15. Depression	1 item per subscale	Not applicable
**Bradley et al.** [58] Demirci et al. [59]	Audit of Diabetes Dependent Quality of Life (ADDQOL)-13	English	Diabetes patients	Quality of life	1. General quality of life 2. Diabetes-specific quality of life 3.Domains impacted by diabetes	1.1 item 2.1 item 3.13 items	Not applicable
Lemon et al. [60]	Audit of Diabetes Dependent Quality of life (ADDQOL)-16	Spanish	Diabetes patients	Diabetes-specific quality of life	1. General quality of life 2. Diabetes-specific quality of life 3. Domains impacted by diabetes	1.1 item 2.1 item 3.16 items	Not applicable
**Speight et al.** [61]	Audit of Diabetes Dependent Quality of life (ADDQOL) 17-senior	English	Diabetes patients	Quality of life	1. General quality of life 2. Diabetes-specific quality of life 3. Domains impacted by diabetes	1.1 item 2.1 item 3.17 items	Not applicable
Costa et al. [62] Daher et al. [63] Kamarul Imran [64]	Audit of Diabetes Dependent Quality of Life (ADDQOL)-18	Portuguese	Diabetes patients	Quality of life	1. General quality of life 2. Diabetes-specific quality of life 3. Domains impacted by diabetes	1.1 item 2.1 item 3.18 items	Not applicable
Abbatecola et al. [65] Bak et al. [66] Fung et al. [67] Jannoo et al. [68] Kong et al. [69] Magwood et al. [70] Soon et al. [71] Turk et al. [72] Visockiene et al. [73] Wee et al. [74] Zhang et al. [75]	Audit of Diabetes Dependent Quality of Life (ADDQOL)- 19	English	Diabetes patients	Quality of life	1. General quality of life 2.Diabetes-specific quality of life 3. Domains impacted by diabetes	1.1 item 2.1 item 3.19 items	Not applicable
**Elasy et al.** [76]	A health status instrument developed for South-African women	English	Women with type 2 diabetes	Health-related quality of life	1. Mental well-being 2. Social well-being	24 items in total	Not applicable
**Hayes et al.** [77] Hayes et al. [78]	Ability to Perform Physical Activities of Daily Living (APPADL)	English	Type 2 diabetes patients and obesity	Impact of weight on activities of daily living	Ability to perform daily physical activities	7 items in total	The APPADL is the renamed version of the Impact of Weight on Activities of Daily Living questionnaire (IWADL)
Torres et al. [79] **Welch et al.** [80]	Attitudes to Diabetes (ATT)-19	English	Diabetes patients	Psychological adjustment to diabetes	Psychological adjustment to diabetes	19 items in total	Torres et al. [79] reports 19 items that include six factors Welch et al. [80] reports a 19-item single subscale
Dunn et al. [81]	Attitude to Diabetes (ATT)-39	English	Diabetes patients	Psychological adjustment to diabetes	1. Stress 2. Adaptation 3. Guilt 4. Alienation 5. Illness conviction 6. Tolerance for ambiguity	39 items in total	Not applicable
Ting et al. [82]	Chinese Diabetes Distress Screening (CDDS)-15	Chinese	Diabetes patients	Diabetes distress	1. Emotional burden 2. Regimen- and social support related distress 3. Physician-related distress	1.6 items 2.6 items 3.3 items	Not applicable
Carter et al. [83] Lehmann et al. [84] McHale et al. [85] Rankin et al. [86] Zhang et al. [87]	Centre for Epidemiological Studies Depression scale (CESD)	English	General population	Depression	1. Depressed affect 2. Positive affect 3. Somatic 4. Interpersonal	1.7 items 2.4 items 3.7 items 4.2 items	Lehmann et al. [84] reports that the PROM consists of 20 items in total
Hsu et al. [88]	Clinically Useful Depression Outcome Scale (CUDOS)	English	Mental disorders	Depression Depressive symptoms	1. Symptoms of depression 2. Psychosocial disabilities 3. Effect of depression on quality of life	1.16 items 2.1 item 3.1 item	Not applicable
**Price et al.** [89] Jaksa et al. [90] Fagerdahl et al. [91] Sriyani et al. [92] Granado-Casas et al. [93]	Cardiff Wound Impact Schedule (CWIS)	English	Diabetes patients	Quality of life in persons with chronic wounds	1. Social life 2. Well-being 3. Physical symptoms and everyday living 4. Quality of life	1.7 items 2.7 items 3.12 items	Not applicable
Huang et al. [94]	Chinese Cardiff Wound Impact Schedule (CCWIS)	Chinese	Diabetes patients	Quality of life in persons with chronic wounds	1. Social life 2. Well-being 3. Physical symptoms and everyday living	1.12 items 2.6 items 3.12 items	Not applicable
**Boyer et al.** [95] Hirsch et al. [48] Huang et al. [50] Khader et al. [96] Lopez-Carmona et al. [97] Nguyen et al. [98] Queiroz et al. [99]	Diabetes-39 (D-39)	English	Diabetes patients	Quality of life	1. Energy and mobility 2. Diabetes control 3. Anxiety and worry 4. Social burden 5. Sexual functioning	1.15 items 2.12 items 3.4 items 4.5 items 5.3 items	Not applicable
**Leite et al. **[100]	Diabetes-39 scale (D-39) Short Form	English	Diabetes patients	Health-related quality of life	1. Energy and mobility 2. Diabetes control 3. Anxiety and worry 4. Social burden 5. Sexual functioning	1.5 items 2.5 items 3.4 items 4.5 items 5.3 items	Not applicable
Anderson et al. [29] **Fitzgerald et al. **[30] Li et al. [101]	Diabetes Care Profile (DCP)	English	Diabetes patients	Diabetes-specific measure of self-care Diabetes-related quality of life	1. Control problems 2. Social and personal factors 3. Positive attitude 4. Negative attitude 5. Self-care ability 6. Importance of care 7. Self-care adherence 8. Diet adherence 9. Medical barriers 10. Exercise barriers 11. Monitoring barriers 12. Understanding mgt. practice 13. Long-term care benefits 14. Support attitudes	1.18 items 2.13 items 3.5 items 4.6 items 5.4 items 6.4 items 7.4 items 8.4 items 9.8 items 10.5 items 11.11 items 12.10 items 13.5 items 14.6 items	Anderson et al. [29] validates a subsample of the scales
Sousa et al. [102] Zauszniewski et al. [103]	Depressive Cognition Scale (DCS)	English	Older adults	Identifying depressive cognitions	Depressive cognition	8 items in total	Not applicable
**Sato et al. **[104]	Diabetes Diet-Related Quality of Life (DDRQOL) Scale	Japanese	Type 2 diabetes patients	Diabetes diet-related quality of life	1. Satisfaction with diet 2. Burden of diet therapy 3. Perceived merits of diet therapy 4. General perception of diet 5. Restriction of social functions 6. Vitality 7. Mental health	1.4 items 2.8 items 3.5 items 4.1 item 5.2 items 6.4 items 7.5 items	Not applicable
**Sato et al. **[105]	Diabetes Diet-Related Quality of Life (DDRQOL)-R	Japanese	Type 2 diabetes patients	Diabetes diet-related quality of life	1. Satisfaction with diet 2. Burden of diet therapy 3. Perceived merits of diet therapy	1.4 items 2.8 items 3.5 items	Not applicable
**Sato et al. **[105]	Diabetes Diet-Related Quality of Life (DDRQOL)-R Short Form	Japanese	Type 2 diabetes patients	Diabetes diet-related quality of life	1. Satisfaction with diet 2. Burden of diet therapy 3. Perceived merits of diet therapy	1.3 items 2.3 items 3.3 items	Not applicable
**Fisher et al. **[106]	Brief Diabetes Distress Screening (DDS)-2	English Spanish	Type 2 diabetes patients	Diabetes distress	Diabetes distress	2 items in total	The DDS-2 was derived from the DDS-17 questionnaire
Chew et al. [107] Chin et al. [108] Curcio et al. [109] Farm et al. [110] Fenwick et al. [111] Graue, 2012 [112] Martinez-Vega et al. [113] Mocan and Bāban [114] **Polonsky et al. **[115] Batais et al. [116] Krzemińska et al. [117]	17-item Diabetes Distress Scale (DDS-17)	English	Diabetes patients	Diabetes distress	1. Emotional burden 2. Physician-related distress 3. Regimen-related distress 4. Interpersonal distress	1.5 items 2.4 items 3.5 items 4.3 items	Not applicable
Thanakwang et al. [118]	Diabetes Distress Scale (DDS)-Thai	Thai	Elderly diabetes patients	Diabetes distress	1. Emotional and regimen-related burden 2. Physician- and nurse-related distress 3. Diabetes-related interpersonal distress	1.10 items 2.4 items 3.3 items	Not applicable
**Batais et al. **[116]	Diabetes Distress Scale (DDS)-Saudi-Arabian	Saudi-Arabian	Type 2 Diabetes patients	Diabetes distress	1. Emotional burden 2. Physician-related distress 3. Regimen-related distress 4. Interpersonal distress	1.5 items 2.4 items 3.5 items 4.3 items	Not applicable
**Kokoszka et al. **[119]	Depression in Diabetes Self-Rating Scale	Polish	Diabetes patients	Depressive symptoms	Depressive symptoms	6 items in total	Not applicable
Poole et al. [120]	Dreiser's Functional Hand Index (DFI)	French	Osteoarthritis	Hand function	Difficulties of ten different daily activities to execute	10 items in total	The DFI Is also called the functional index for hand osteoarthritis (FIHOA)
**Abetz et al. **[31]	Diabetes Foot Ulcer Scale (DFS)	English	Diabetes patients	Impact of foot ulcers and their treatment on quality of life	1. Leisure 2. Physical health 3. Daily activities 4. Emotions 5. Non-compliance 6. Family 7. Friends 8. Positive attitude 9. Treatment 10. Satisfaction 11. Financial	1.5 items 2.6 items 3.6 items 4.17 items 5.2 items 6.5 items 7.5 items 8.5 items 9.4 items 10.1 item 11.2 items	Not applicable
**Bann et al. **[121] Hui et al. [122] Macioch et al. [123] Martinez-Gonzalez et al. [124] Kontodimopoulos et al. [125]	Diabetes Foot Ulcer Scale (DFS-SF)	English	Diabetes patients	Impact of diabetic foot ulcers on patients’ quality of life	1. Leisure 2. Physical health 3. Worried about ulcers 4. Dependence/ daily life 5. Negative emotions 6. Bothered by ulcer care	1.5 items 2.5 items 3.4 items 4.5 items 5.6 items 6.4 items	Macioch et al. [123] uses a different number of items per subscale after translation and adaption of the Polish version
Meadows et al. [126] Mulhern et al. [127] **Mulhern et al. **[128] Tan et al. [129] Benazizi et al. [130]	Diabetes Health Profile (DHP)-18	English	Diabetes patients	Psychological and behavioural impact that diabetes can have on a person's daily live due to diabetes	1. Psychological distress 2. Barriers to activity 3. Disinhibited eating	1.6 items 2.7 items 3.5 items	Mulhern et al. [128] developed a Diabetes Health Profile-3 Dimension and Diabetes Health Profile-5 Dimension based on the DHP-18 Tan et al. [129] validates a subscale of the DHP-18
Goddijn et al. [131]	Diabetes Health Profile (DHP)-31	Dutch	Diabetes patients	Psychological and behavioural impact that diabetes can have on a person's daily live due to diabetes	1. Psychological distress 2. Barriers to activity 3. Disinhibited eating	1.14 items 2.12 items 3.5 items	Goddijn et al. [131] deleted an item for the analysis
Holmes-Truscott et al. [32]	DAWN2 Impact of Diabetes Profile (DIDP)-6	English	Diabetes patients	Perceived impact of diabetes on quality of life	1. Physical health 2. Financial situation 3. Relationships 4. Leisure activities 5. Work or studies 6. Emotional well-being	1 item per subscale	Not applicable
Holmes-Truscott et al. [32]	DAWN2 Impact of Diabetes Profile (DIDP)-7	English	Diabetes patients	Perceived impact of diabetes on quality of life	1. Physical health 2. Financial situation 3. Relationships 4. Leisure activities 5. Work or studies 6.Emotional well-being 7.Dietary freedom	1 item per subscale	Not applicable
**Hammond et al. **[132] Li et al. [133]	Diabetes Impact Measurement Scales (DIMS)	English	Diabetes patients	Health status	1.Symptoms 2.Well-being 3. Diabetes-related morale 4. Social role fulfillment	1.17 items 2.11 items 3.11 items 4.5 items	Not applicable
**Lin et al. **[134] Saffari et al. [135]	Diabetes-Specific Quality of Life Questionnaire (DMQoL)	Mandarin	Diabetes patients	Health-related quality of life	Health-related quality of life	10 items in total	Not applicable
**Shen et al. **[35]	Diabetes Quality of Life Clinical Trial Questionnaire (DQLCTQ)	English	Diabetes patients	Quality of life of diabetes patients in clinical trials	*Generic* 1. General health 2. Comparative health 3. Physical functioning 4. Global role functioning 5. Social functioning 6. General social functioning 7. Energy/fatigue 8. Health distress 9. Mental health *Diabetes-specific DQOL* 1. Satisfaction 2. Impact 3. Social worry 4. Diabetes worry *Worry* *Newly developed* 1. Treatment satisfaction 2. Treatment flexibility 3. Social stigma 4. Frequency of symptoms 5. Bothersomeness of symptoms 6. Self-efficacy *Demographics*	*Generic* 1.1 item 2.1 item 3.6 items 4.2 items 5.1 item 6.1 item 7.5 items 8.6 items 9.5 items *Diabetes-specific DQOL* 1.18 items 2.27 items 3.7 items 4.7 items *Worry* 17 items *Newly developed* 1.3 items 2.10 items 3.4 items 4.7 items 5.7 items 6.3 items *Demographics* 4 items	Not applicable
**Shen et al. **[35]	Diabetes Quality of Life Clinical Trial Questionnaire-Revised (DQLCTQ-Rev)	English	Diabetes patients	Quality of life of diabetes patients in clinical trials	1. Physical function 2. Energy/fatigue 3. Health distress 4. Mental health 5. Satisfaction 6. Treatment satisfaction 7. Treatment flexibility 8. Frequency of symptoms	57 items in total	Not applicable
**Goh et al. **[33]	Asian Diabetes Quality of Life (DQOL)-Chinese-18	Mandarin	Diabetes patients	Diabetes-specific quality of life	1. Financial concerns 2. Relationship 3. Memory 4. Diet and activities 5. Energy levels	18 items in total	Not applicable
**Goh et al. **[33] Permana et al. [136]	Asian Diabetes Quality of Life (DQOL)-English-21	English	Diabetes patients	Diabetes-specific quality of life	1. Financial 2. Energy levels 3. Memory and cognition 4. Relationship 5. Diet	21 items in total	Not applicable
**Goh et al. **[33]	Asian Diabetes Quality of Life (DQOL)-Malay-21	Malay	Diabetes patients	Diabetes-specific quality of life	1. Financial 2. Energy levels 3. Memory and cognition 4. Relationship 5. Diet	21 items in total	Not applicable
**Burroughs et al. **[137] Dudzinska et al. [138] Magwood et al. [70] Samah et al. [139] Tang et al. [140]	Diabetes Quality of Life (DQOL-15)	English	Diabetes patients	(Diabetes-specific) quality of life	1. Satisfaction 2. Impact 3. Social/worry 4. Vocational/worry	1.5 items 2.4 items 3.4 items 4.2 items	Dudzinska et al. [138] mentions 15 items in total but no specific domains The DQOL-42 is also called the Diabetes Quality of Life (DQOL)-BCI
**Diriba et al. **[141]	Diabetes Quality of Life (DQOL)- Afaan Oromoo-34	Afaan Oromoo	Type 2 diabetes	Diabetes-related quality of life	1. Satisfaction 2. Impact 3. Social/worry 4. Vocational/worry	1.13 items 2.13 items 3.5 items 4.3 items	Not applicable
**Cheng et al. **[142] Cheng et al. [143] Huang et al. [144]	Diabetes Quality of Life (DQOL)-42	Chinese	Elderly diabetes patients	Quality of life	1. Satisfaction 2. Impact 3. Diabetes-related worry	1.15 items 2.20 items 3.7 items	Cheng et al. [143] only reports that the PROM consists of 42 items in total
Yildirim et al. [145]	Diabetes Quality of Life (DQOL)-45	Turkish	Diabetes patients	(Diabetes-specific) quality of life	1. Satisfaction 2. Impact 3. Diabetes-related worry 4. Social/vocational worry	45 items in total	Not applicable
Jacobson et al. [51] Pakpour et al. [146] Sato et al. [147] Rankin et al. [148]	Diabetes Quality of Life (DQOL)-46	English	Diabetes patients	(Diabetes-specific) quality of life	1. Satisfaction 2. Impact 3. Diabetes-related worry 4. Social/vocational worry	1.15 items 2.20 items 3.4 items 4.7 items	Not applicable
Bujang et al. [149]	Diabetes Quality of Life (DQOL)-60	Malay	Diabetes patients	(Diabetes-specific) quality of life	*Diabetes* 1. Diabetes life satisfaction scale 2. Disease impact scale 3. Disease related worries scale *General health questionnaire*	*Diabetes* 1.18 items 2.27 items 3.14 items *General health questionnaire* 1 item	Not applicable
**Bujang et al. **[150]	Diabetes Quality of Life (DQOL)-revised version	Malay	Diabetes patients	(Diabetes-specific) quality of life	1. Diabetes life satisfaction scale 2. Disease impact scale 3.Disease related worries scale	1.6 items 2.4 items 3.3 items	Not applicable
Correr et al. [151]	Diabetes Quality of Life (DQOL)-Brazil	Brazilian	Diabetes patients	(Diabetes-specific) quality of life	1. Satisfaction 2. Impact 3. Concern: social/vocational 4. Concern: related to diabetes	1.15 items 2.18 items 3.7 items 4.4 items	Correr et al. [151] performs the intercultural translation of the DQOL-Brazil to Portuguese
**Brasil et al. **[152]	Diabetes Quality of Life (DQOL)-Brazil-8	Brazilian	Diabetes patients	Health-related quality of life	1. Satisfaction 2. Impact 3. Concern: social/vocational 4. Concern: related to diabetes	1.2 items 2.3 items 3.1 item 4.2 items	Not applicable
**Jin et al. **[153]	Diabetes Quality of Life (DQOL)-Chinese-24	Chinese	Diabetes patients	Quality of life	1. Satisfaction 2. Impact 3. Worry	1.9 items 2.8 items 3.7 items	Not applicable
**Al-Qerem et al. **[154]	Diabetes Quality of Life (DQOL)-Arabic-29	Arabic	Type 2 Diabetes patients	Quality of life	1. Satisfaction 2. Impact 3. Worry	1.14 items 2.11 items 3.4 items	Not applicable
Millán et al. [155]	Diabetes Quality of Life (DQOL)-Spanish-43	Spanish	Diabetes patients	Relative burden of an intensive diabetes treatment regimen	1. Satisfaction 2. Impact 3. Concern: social/vocational 4. Concern: related to diabetes	1.15 items 2.17 items 3.7 items 4.4 items	Not applicable
**Alavi et al. **[156] Jahanlou et al. [157]	Iranian Diabetes Quality of Life (IRDQOL)-41	Farsi	Diabetes patients	General- and health-related quality of life	1. General quality of life 2. Health-related quality of life	41 items in total [13 general quality of life items]	Not applicable
**Lee et al. **[158]	Diabetes-specific Quality of Life scale (D-QOL)-34	Korean	Diabetes patients	Health-related quality of life Depression Distress	1. Emotional suffering 2. Social functioning 3. Adherence to treatment 4. Diabetes-specific items	24 items in total	Not applicable
**Grootenhuis et al. **[159]	Type 2 Diabetes Symptom Checklist (DSC)	Dutch	Type 2 diabetes patients	Diabetes symptom severity and changes over time	1. Psychological fatigue 2. Psychological cognitive 3. Neuropathic pain 4. Neuropathic sensoric 5. Cardiovascular 6. Vision 7. Hypoglycemic 8. Hyperglycemic	1.4 items 2.4 items 3.4 items 4.6 items 5.4 items 6.5 items 7.3 items 8.4 items	Not applicable
Arbuckle et al. [160] Naegeli et al. [161]	Diabetes Symptom Checklist-Revised (DSC-R)	Dutch	Diabetes patients	Symptom burden of diabetes	1. Psychological fatigue 2. Psychological cognitive 3. Neuropathic pain 4. Neuropathic sensoric 5. Cardiovascular 6. Ophthalmologic 7. Hypoglycemic 8.H yperglycemic	1.4 items 2.4 items 3.4 items 4.6 items 5.4 items 6.5 items 7.3 items 8.4 items	Naegeli et al. [161] validates a subset of items
Lee et al. [162]	Korean- Diabetes Symptom Checklist- Revised (K-DSC-R)	Korean	Type 2 diabetes patients	Symptom burden of diabetes and its possible complications	1. Neuropathic pain 2. Psychological fatigue 3. Hypoglycemic 4. Ophthalmologic 5. Hyperglycemic 6. Cardiovascular 7. Sensory neuropathic	1.6 items 2.5 items 3.5 items 4.4 items 5.4 items 6.3 items 7.2 items	Not applicable
**Garcia et al. **[26]	Diabetes Symptom Self-Care Inventory (DSSCI)	English Spanish	Diabetes patients	Diabetes symptoms and actions in response to those symptoms	Diabetes symptoms	At least 48 items, including a list of 38 symptoms. The remaining items refer to attitudes and actions taken for specific symptoms	The DSSCI is a decision tree rather than a scale
**Araki et al. **[36]	Elderly Diabetes Burden Scale (EDBS)	Japanese	Elderly diabetes patients	Diabetes-specific and non-specific quality of life	1. Symptom burden 2. Social burden 3. Dietary restrictions 4. Worry 5. about diabetes 6. Treatment (dis)-satisfaction 7. Burden by tablets or insulin	1.4 items 2.5 items 3.4 items 4.4 items 5.3 items 6.3 items	Not applicable
de Cock et al. [163]	Edinburgh Depression Scale (EDS)	English	Postnatal women	Screening depression	Depression	10 items in total	The EDS is the renamed version of the Edinburgh Postnatal Depression Scale
Clarke et al. [164] Ekwunife et al. [165] Glasziou et al. [47] Konerding et al. [166] Lee et al. [167] Luo et al. [168] Matza et al. [169] Mulhern et al. [127] Pan et al. [170] Pattanaphesaj et al. [171] Turk et al. [72] Wang et al. [172] Yordanova et al. [55] Arifin et al. [173] Zare et al. [174] Janssen et al. [175]	EuroQol (EQ)-5D-3L	Dutch Finnish Norwegian Swedish English	General population	Generic measure of health status Health-related quality of life	1. Mobility 2. Self-care 3. Usual activities 4. Pain/discomfort 5. Anxiety/depression	1 item per subscale	Not applicable
**Koh et al. **[176] Matza et al. [177] Pan et al. [170] Pattanaphesaj et al. [171] Sayah et al. [178] Wang et al. [179] Wang et al. [172] Arifin et al. [173] Janssen et al. [175]	EuroQol (EQ)-5D-5L	Dutch Finnish Norwegian Swedish English	General population	Generic measure of health status Health-related quality of life	1. Mobility 2. Self-care 3. Usual activities 4. Pain/discomfort 5. Anxiety/depression	1 item per subscale	Not applicable
Cinar et al. [180]	13-item Fatigue subscale of the FACIT-F	English	Chronic Diseases and Generic for Neoplasms	Fatigue	Fatigue	13 items in total	Not applicable
Leonardson et al. [181]	General well-being schedule	English	General population	Subjective feelings of psychological well-being and distress	1. Self-esteem 2. Depression 3. Vitality 4. Health concerns	1.8 items 2.6 items 3.4 items 4.4 items	Not applicable
Amidu et al. [182]	Golombok-Rust Inventory of Sexual Satisfaction (GRISS)	English	Heterosexual couples or individuals who have a current heterosexual relationship	Sexual functioning	*Male* 1. Impotence 2. Premature ejaculation 3. Nonsensuality 4. Avoidance 5. Dissatisfaction 6. Infrequency 7. Noncommunication *Female* 1. Vaginismus 2.Anorgasmia 3. Nonsensuality 4. Avoidance 5. Dissatisfaction 6. Infrequency 7. Noncommunication	4 items per subscale	Not applicable
Poole et al. [120]	Hand Function Disability Scale (HFDS)	French	Rheumatoid arthritis	Hand function	1. Kitchen 2. Dressing 3. Hygiene 4. Office 5. Other	1.8 items 2.2 items 3.2 items 4.2 items 5.4 items	The HFDS is also called Cochin Scale or Duruoz’s Hand Index
Hajos et al. [183]	the Worry subscale from the Hypoglycemia Fear Survey (HFS-W)	English	Diabetes patients	Fear of hypoglycemia (worry subscale)	Fear of hypoglycemia (worry subscale)	13 items in total	The HFS-W concerns the worry subscale from the Hypoglycemia Fear Survey (HFS)
**Kawata et al. **[184]	Hypoglycemia Perspectives Questionnaire (HPQ)	English	Diabetes patients	Experience and impact of hypoglycemia	1. Symptom concern 2. Compensatory behavior 3. Worry	1.6 items 2.5 items 3.5 items	Not applicable
Morgan et al. [38]	Health Status Questionnaire (HSQ) 2.0	English	General population	Quality of life in several dimensions	1. Health perception 2. Physical functioning 3. Role limitations – physical health 4. Role limitations—emotional problems 5. Social functioning 6. Mental health 7. Bodily pain 8. Energy/fatigue	39 items in total	Not applicable
Maddigan et al. [40]	Health Utilities Index Mark 2 (HUI2)	English	General population	Health-related quality of life Self-reported health status	1. Sensation (hearing, vision and speech) 2. Mobility 3. Emotion 4. Cognition 5. Self-care 6. Pain 7. Fertility	1 item per subscale	Maddigan et al. [40] did not use the ‘Fertility’ subscale
Maddigan et al. [40] Maddigan et al. [185] Mo et al. [186]	Health Utilities Index Mark 3 (HUI3)	English	General population	Health-related quality of life Self-reported health status	1. Vision 2. Hearing 3. Speech 4. Ambulation 5. Dexterity 6. Emotion 7. Cognition 8. Pain/discomfort	1 item per subscale	Not applicable
Kolotkin et al. [187]	Impact of Weight on Quality of Life-Lite (IWQOL-lite)	English	People with obesity	Weight-related quality of life	1. Physical function 2. Self-esteem 3. Sexual life 4. Public distress 5. Work	1.11 items 2.7 items 3.4 items 4.5 items 5.4 items	Not applicable
**Kolotkin et al. **[188] Kolotkin et al. [189]	Impact of Weight on Quality of Life-Lite-Clinical Trials (IWQOL-Lite-CT)	English	People with obesity for obesity clinical trials	Health-related quality of life	1. Physical 2. Psychosocial	1.7 items 2.13 items	Kolotkin et al. [188] validates the pilot version of the IWQOL-Lite-CT which consists of 22 items
Tan et al. [129]	Kessler-10 Psychological Distress scale (K10)	English	General population	Level of distress and severity associated with psychological symptoms	1. Depressed mood 2.Motor agitation 3. Fatigue 4. Worthless guilt 5. Anxiety	1.3 items 2.2 items 3.2 items 4.1 item 5.2 items	Not applicable
Hirsch et al. [48]	LQD Quality of Life with Diabetes (LQD)	German	Diabetes patients	Diabetes-specific quality of life	1. Diabetes satisfaction 2. Diabetes stress 3. Blood glucose stress	1.7 items 2.7 items 3.3 items	Not applicable
Hasan et al. [190]	Menopause-specific Quality of Life (MENQOL)	English	Menopause	Quality of life	1.Vasomotor 2.Physical 3.Psychosocial 4.Sexual functioning	1.3 items 2.7 items 3.16 items 4.3 items	Not applicable
Poole et al. [120]	Michigan Hand Outcomes Questionnaire (MHQ)	English	Patients with hand disorders	Hand-specific outcome measure	1. Overall hand function 2. Activities of daily living 3. Work performance 4. Pain 5. Aesthetics 6. Satisfaction with hand function	1.10 items 2.17 items 3.5 items 4.10 items 5.8 items 6.12 items	Not applicable
Kim et al. [191]	MOS Six-Item Sleep Scale	English	General population	Quality of sleep	Not clearly reported, but includes at least the following dimensions of sleep 1. Initiation 2. Maintenance 3. Adequacy 4. Somnolence 5. Respiratory impairments	6 items in total	Not applicable
**Borg et al. **[192] Svedbo Engstrom et al. [193] Svedbo Engstrom et al. [194]	National Diabetes Register (NDR) survey	Swedish	Diabetes patients	Patient well-being, abilities to manage diabetes and judgements of their experience with diabetes care	1. How you feel 2. our worries 3. Your capabilities to manage your diabetes 4. Barriers 5. Support from others 6. Support from diabetes care providers 7. Medical devices and medical treatment	1.5 items 2.3 items 3.5 items 4.5 items 5.3 items 6.9 items 7.3 items	Svedbo Engstrom et al. [193] validates a pilot version of the NDR
**Vileikyte et al. **[195]	Neuropathy- and Foot Ulcer–Specific Quality of Life instrument (NeuroQoL)	English	Patients with diabetic peripheral neuropathy	Neuropathy- and foot ulcer- specific quality of life	*Physical symptoms* 1. Pain 2. Reduced feeling 3. Diffuse sensory motor *Psychosocial symptoms* 1. Interpersonal/ emotional burden 2. Activity limitations	*Physical symptoms* 1.7 items 2.3 items 3.3 items *Psychosocial symptoms* 1.11 items 2.3 items	Not applicable
Keinanen et al. [196]	Nottingham Health Profile (NHP)	English	General population	Health-related quality of life	*Current Health-related Quality of Life* 1. Energy 2. Sleep 3. Pain 4. Emotional reactions 5. Social isolation 6. Physical mobility *Health problems* 1. Paid employment 2. Jobs around the house 3. Social life 4. Home life 5. Sex life 6. Hobbies 7. Holidays	*Current health-related quality of life* 1.3 items 2.5 items 3.8 items 4.9 items 5.5 items 6.8 items *Health problems* 7 items in total	Not applicable
McGuire et al. [197]	Short Form Problem Areas in Diabetes (PAID)-1	English	Diabetes patients	Emotional impact of diabetes	Emotional problems	1 item in total	Not applicable
Lee et al. [198] McGuire et al. [197] Vislapuu et al. [199]	Problem areas in diabetes (PAID)-5	English	Diabetes patients	Diabetes-related emotional functioning	Emotional problems	5 items in total	Not applicable
Siaw et al. [200] Venkataraman et al. [201]	Problem areas in diabetes (PAID)-16	English	Diabetes patients	Diabetes-specific emotional distress	1. Emotional and management problems 2. Diabetes problems 3. Support problems	1.11 items 2.3 items 3.2 items	Not applicable
Arzaghi et al. [202] Belendez et al. [203] **Eom et al. **[204] Fenwick et al. [111] Graue et al. [112] Gross et al. [205] Huang et al. [206] Huis In’t Veld et al. [207] Jannoo et al. [208] Miller et al. [209] Papathanasiou et al. [210] Polonsky et al. [211] Snoek et al. [212] Tan et al. [129] Venkataraman et al. [201] Welch et al. [213] Welch et al. [214] Welch et al. [215] Cichoń et al. [216]	Problem areas in diabetes (PAID)-20	English	Diabetes patients	Diabetes-related emotional functioning Distress	1. Emotional problems 2. Treatment problems 3. Food-related problems 4. Social support problems	1.12 items 2.2 items 3.3 items 4.3 items	Not applicable
Matza et al. [169]	Psychological General Well-Being Index (PGWB)	English	General population	Subjective feelings of psychological well-being and distress	1. Anxiety 2. Depressed mood 3. Positive well-being 4. Self-control 5. General health 6. Vitality	1.5 items 2.3 items 3.4 items 4.3 items 5.3 items 6.4 items	Not applicable
**Chuayruang et al. **[28]	Patient-reported outcomes in Thai patients with type 2 diabetes mellitus (PRO-DM-Thai)	Thai	Type 2 diabetes patients	Patient-reported outcomes (outcomes of diabetes care)	1. Physical function 2. Symptoms 3. Psychological well-being 4. Self-care management 5. Social well-being 6. Global judgements of health 7. Satisfaction with care and flexibility of treatment	1.5 items 2.7 items 3.5 items 4.12 items 5.5 items 6.5 items 7.5 items	Not applicable
Zhu et al. [217]	Pittsburg Sleep Quality Index (PSQI)	English	Adults	Sleep quality	1. Subjective sleep quality 2. Sleep latency 3. Sleep duration 4. Habitual sleep efficiency 5. Sleep disturbances 6. Use of sleep medication 7. Daytime dysfunction	1.1 item 2.2 items 3.1 item 4.3 items 5.9 items 6.1 item 7.2 items	Zhu et al. [217] removed ‘use of sleep medication’ from the PSQI questionnaire
Oobe et al. [37]	Quality of life (QOL) questionnaire	Japanese	Diabetes patients	Quality of life	1. Degree of apprehension 2. Degree of distress 3. Degree of satisfaction with life 4. Degree of satisfaction with treatments	1.5 items 2.5 items 3.4 items 4.4 items	Not applicable
**Orozco-Beltran et al. **[218]	Impact of hypoglycemia on the HRQoL of type 2 diabetes patients (QoLHYPO©) questionnaire	Spanish	Type 2 diabetes patients	Quality of life	Health-related quality of life	13 items in total	Not applicable
**Nagpal et al. **[34]	Quality of Life for Indian diabetes Patients (QOLID)	Indian	Type 2 diabetes patients	Quality of life	1. Social life, work and travel 2. Physical endurance 3. General health 4. Treatment satisfaction 5. Symptom botherness 6. Financial worries 7. Emotional/mental health 8. Diet advise tolerance	1.6 items 2.6 items 3.3 items 4.4 items 5.3 items 6.4 items 7.5 items 8.3 items	Not applicable
**Mikhael et al. **[219]	Quality of life scale for Iraqi Diabetes patients (QOLSID)	Irak	Diabetes patients	Quality of life	1. Satisfaction 2. Stress	1.8 itmes 2.2 items	Not applicable
Whitty et al. [220]	Self-completion instrument for diabetes	English	Diabetes patients	Subjective health status	The instrument consists of six multi-item scales 1. Physical function and energy 2. Depression 3. Psychological distress and barriers to activity 4. Symptoms	1.Physical function 10 items and Energy 4 items 2.7 items 3.Psychological distress 16 items and Barriers to activity 15 items 4.9 items	Whitty et al. [220] reduced the ‘Symptoms’ scale to 9 items for analysis. The ‘Symptoms’ scale originally consists of 20 items
**Rao et al. **[39]	Self-perception of health	English	Type 2 diabetes patients	Subjective feelings of psychological well-being and distress	1. Positive self-feeling 2. Sociality 3. Attention seeking 4. Feel healthy 5. Worry about health 6. Dependence	1.6 items 2.6 items 3.4 items 4.5 items 5.2 items 6.4 items	Not applicable
Maddigan et al. [40] Maurischat et al. [41] Wan et al. [42]	12-Item Short Form Health Survey (SF-12)	English	General population	Health-related quality of life	1. Physical functioning 2. Role physical 3. Role emotional 4. Pain 5. Vitality 6. General health 7. Social functioning 8. Mental health	1.2 items 2.2 items 3.2 items 4.1 item 5.1 item 6.1 item 7.1 item 8.2 items	Not applicable
Westaway et al. [43]	20-item Short Form Health Survey (SF-20)	English	General patient population	Health-related quality of life	1. Physical functioning 2. Role functioning 3. Social functioning 4. Mental health 5. General health 6. Bodily pain	1.6 items 2.2 items 3.1 item 4.5 items 5.5 items 6.1 item	Not applicable
Ahroni et al. [45] Anderson et al. [29] Bagheri et al. [46] Glasziou et al. [47] Hirsch et al. [48] Hu et al. [49] Huang et al. [50] Jacobson et al. [51] Linzer et al. [52] Martin et al. [53] Woodcock et al. [54] Yordanova et al. [55] Abbasi-Ghahramanloo et al. [44]	36-Item Short Form Health Survey (SF-36)	English	General population	Generic health concepts Health-related quality of life	1. Physical functioning 2. Social functioning 3. Role limitations due to physical problems 4. Role limitation due to emotional problems 5. Mental health 6. Energy and vitality 7. Pain 8. General perception of health 9. Perceived change in health	1.10 items 2.2 items 3.4 items 4.3 items 5.5 items 6.4 items 7.2 items 8.5 items 9.1 item	Ahroni et al. [45], Bagheri et al. [46], Jacobson et al. [51] and Martin et al. [53] analyzed specific subscales of the SF-36 questionnaire
**Hsu et al. **[221]	Short Form Problem Areas in Diabetes in chinese (SF-PAID-C)	Chinese	Diabetes patients	Diabetes-specific emotional distress	1. Diabetes-related emotional problems 2. Problems related to food	1.6 items 2.2 items	Not applicable
Wicaksana et al. [222]	Patient Health Questionnaire (PHQ)-4	English	General population	Psychological distress	1. Depression 2. Anxiety	1.2 items 2.2 items	Not applicable
Lupascu et al. [223] Twist et al. [224] Udedi et al. [225] Zhang et al. [226]	Patient Health Questionnaire (PHQ)-9	English	General population	Depressive symptoms Depression	Depressive symptoms/ depression	9 items in total	Not applicable
Klis et al. [27]	Pictorial Representation of Illness and Self Measure Revised II (PRISM-RII)	Dutch	Diabetes patients	Diabetes-related suffering	1. Self illness separation (SIS) 2.Illness perception measure	Not applicable	The PRISM-RII is a visual interactive PROM
Duran et al. [227]	Questionnaire on Stress in Diabetic Patients (QSD)	German	Diabetes patients	Diabetes distress	1. Fear of long-term complications 2. Dietary restrictions 3. Problems with hypoglycemia 4. Difficulties with treatment regimen 5. Problems with acceptance 6. Reduction of performance 7. Problems with work 8. Strained doctor-patient relationship 9. Problems with relationship or family 10. Feeling patronized	1.7 items 2.4 items 3.9 items 4.10 items 5.15 items 6.11items 7.6 items 8.5 items 9.12 items 10.6 items	Not applicable
Herschbach et al. [228]	Questionnaire on Stress in Patients with Diabetes-Revised (QSD-R)	German	Diabetes patients	Diabetes distress	1. Leisure time 2. Depression/fear of future 3. Hypoglycemia 4. Treatment regimen/diet 5. Physical complaints 6. Work 7. Partner 8. Doctor-patient relationship	1.4 items 2.6 items 3.4 items 4.9 items 5.6 items 6.6 items 7.6 items 8.4 items	Not applicable
Kinik and Çamlicali [229]	Questionnaire on Stress in Patients with Diabetes-Revised-Turkish (QSD-R-TR)	Turkish	Diabetes patients	(Diabetes) distress	1. Leisure time 2. Work 3. Relationship with partner 4. Doctor-patient relationship 5. Problems with hypoglycemia 6. Treatment regimen 7. Physical complaints 8. Worries about long-term complications	1.4 items 2.6 items 3.6 items 4.4 items 5.4 items 6.7 items 7.6 items 8.6 items	Not applicable
Fraim et al. [230]	Questionnaire on Stress in Patients with Diabetes-Revised-Turkish-Cyprus (QSD-R-TR)	Turkish	Diabetes patients	(Diabetes) distress	1. Psycho-physiological aspects 2. Social 3. Accountability 4. Psychosocial distress 5. Fear/depression 6. Outcomes 7. Medical relationships	1.11 items 2.6 items 3.6 items 4.4 items 5.4 items 6.3 items 7.4 items	Not applicable
Pakpour et al. [231]	Sexual Quality of Life questionnaire-Female (SQOL-F)	English	Women with sexual dysfunction	Impact of sexual dysfunction on quality of life	Impact of sexual dysfunction on quality of life	18 items in total	Not applicable
**Polonsky et al. **[232]	Type 2 Diabetes Distress Assesment System (T2-DDAS)	English	Adults with T2D	Diabetes Distress	1. Core distress 2. Management demands 3. Hypoglycemia 4. Long-term health 5. Health care provider 6. Interpersonal issues 7. Shame 8. Healthcare access	1.8 items 2.3 items 3.3 items 4.3 items 5.3 items 6.3 items 7.3 items 8.3 items	Not applicable
Pouwer et al. [233] Pouwer et al. [234]	Well-being questionnaire (W-BQ) 12	Dutch	Patients in clinical trials and other studies	Several aspects of psychological well-being	1. Negative well-being 2. Energy 3. Positive well-being	1.4 items 2.4 items 3.4 items	Not applicable
Hirsch et al. [48] Kolawole et al. [235] Kolawole et al. [24]	Well-being questionnaire (W-BQ) 22	English	Diabetes patients	Well-being Quality of life	1. Depression 2. Anxiety 3. Energy 4. Positive well-being 5. (General well-being)	1.6 items 2.6 items 3.4 items 4.6 items 5.22 items	Not applicable
Speight et al. [236]	Well-being questionnaire (W-BQ) 28	English	Diabetes patients	Well-being Health-related quality of life	1. Generic negative well-being 2. Generic positive well-being 3. Energy 4. Generic stress 5. Diabetes-specific negative well-being 6. Diabetes-specific positive 7. well-being 8. Diabetes-specific stress	1.4 items 2.4 items 3.4 items 4.4 items 5.4 items 6.4 items 7.4 items	Not applicable
Bradley et al. [237]	Well-being scale	English	Diabetes patients	Well-being an treatment satisfaction	1. Depression 2. Anxiety 3. Positive well-being	1.6 items 2.6 items 3.6 items	Not applicable
Mannucci et al. [56]	Well-being Enquiry for Diabetics (WED)	Italian	Diabetes patients	Diabetes-related quality of life	1. Symptoms 2. Discomfort 3. Serenity 4. Impact	1.10 items 2.10 items 3.10 items 4.20 items	Not applicable
Awata et al. [238] Cichón et al. [239] Hajos et al. [240] Halliday et al. [241] Yordanova et al. [55]	The World Health Organisation- Five Well-Being Index (WHO-5)	Danish English	General population	Positive psychological well-being	1. Cheerful and in good spirits 2. Calm and relaxed 3. Active and vigorous 4. Feeling fresh and rested 5. Filled with things that interest me	1 item per subscale	Not applicable
Pibernik-Okanovic et al. [23]	World Health Organisation Quality of Life (WHOQOL-100)	Australian Croatian French Indian Israelic Japanese Dutch Panamees Russian Spanish Thai English Shona	General population	Overall quality of life General health	*Overall quality of life and general health* *Quality of life domains* 1. Physical 2. Psychological 3.Social relationships 4. Environment	*Overall quality of life and general health* Not reported *Quality of life domains* 1.7 facets 2.6 facets 3.3 facets 4.8 facets Each facet consists of 4 questions	Pibernik-Okanovic et al. [23] uses a modified four-domain structure. The standard WHOQOL-100 consists of six domains (i.e. includes domains level of independence and spirituality)
Jahanlou et al. [157] Kolawole et al. [24] Reba et al. [242] Sreedevi et al. [25] Abbasi-Ghahramanloo et al. [44]	World Health Organisation Quality of Life (WHOQOL)-BREF	Australian Croatian French Indian Israelic Japanese Dutch Panamees Russian Spanish Thai English Shona	General population	Quality of life	1. Physical health 2. Psychological 3. Social relationships 4. Environment	1.7 items 2.6 items 3.3 items 4.8 items	Not applicable

**Bold** represents the development paper of the PROM. Not for all of the PROMs a development paper is listed in the table, while those PROMs were not developed in a diabetes population

We identified numerous different versions of the same PROM, for example 17 different versions were identified for the Diabetes Quality of Life questionnaire (DQOL). For many PROMs, these versions arose from translations, which during the validation process were modified by removing items or adding new items. By modifying, this makes it a new PROM, because it cannot be assumed that measurement properties are the same for different versions. When PROMs were only translated, with the same amount of subscales and items per subscales, we tallied this PROM as one of the same version and added the reference to that row of the PROM in Table 1. Finally, two studies consisted of non-standard PROMs, which were a decision tree [26] and a visual interactive PROM [27].

### Levels of HRQOL measured with the PROMs

Table 2 and Supplemental Table 1 provide an overview of the specific levels of HRQOL that the included PROMs measure based on the Wilson and Cleary model [4]. Of the 116 unique HRQOL PROMs, 91 of their subscales measured symptom status, 60 measured functional status and 26 measured general health perceptions. With regard to symptom status, 22/91 measured diabetes-related symptoms, which included problems with vision, hearing, speaking, neuropathy, hypoglycemia, hyperglycemia, motor agitation and vasomotor function disturbance as well as cardiovascular disease. When examining the PROMs, there is overlap between the diabetes-related symptoms subscales and the general symptom status scales referring to physical symptoms and mental symptoms, such as pain or depressive feelings. For example, the Patient-reported outcomes in Thai patients with type 2 diabetes mellitus (PRO-DM-Thai) states to measure diabetes-related symptoms, but these include sleep problems, sexual problems and pain, which could be considered generic symptoms [28].

**Table 2:** Overview of the specific levels of HRQOL that the included PROMs measure based on the Wilson and Cleary model [4]

**PROM**	**Health-related quality of life**												**Other**	
	**Symptom status**							**Functional status**				**General health perception**	**Overall quality of life**	**Characteristics of** **individual/environment or PREM**
	**Diabetes related symptoms**	**Physical symptoms**			**Mental symptoms**			**Physical** **function**		**Psychological function**	**Social function**	**Overall health**	**Overall quality of life**	
		**Pain**	**Energy/ fatigue**	**Sleep**	**Distress**	**Anxiety/worry**	**Depression**	**Activities of daily living**	**Sexual function**	**Emotional function/ cognition**	**Social function/ participation**	**General health perceptions, self-rated health**	**Overall quality of life/well-being**	
15D standardized measure of health-related quality of life Finnish (15D Finnish) [57]	●	●	●	●	●		●	●	●	●				●
Audit of Diabetes Dependent Quality of Life (ADDQOL)-13 [58, 59]													●	
Audit of Diabetes Dependent Quality of life (ADDQOL)-16 [60]													●	
Audit of Diabetes Dependent Quality of life (ADDQOL) 17-senior [61]													●	
Audit of Diabetes Dependent Quality of Life (ADDQOL)-18 [62–64]													●	
Audit of Diabetes Dependent Quality of Life (ADDQOL)- 19 [65–75]													●	
A Health status instrument developed for South-African women [76]										●	●			
The Ability to Perform Physical Activities of Daily Living Questionnaire (APPADL) [77, 78]								●						
Attitudes to Diabetes (ATT)-19 [79, 80]										●				
Attitude to Diabetes (ATT)-39 [81]					●						●			●
Chinese Diabetes Distress screening (CDDS)-15 [82]					●	●	●							
Centre for Epidemiological Studies Depression Scale (CESD) [83–87]							●							
Clinically Useful Depression Outcome Scale (CUDOS) [88]							●			●	●		●	
Cardiff Wound Impact Schedule (CWIS) [89–93]								●			●		●	
Chinese Cardiff Wound Impact Schedule (CCWIS) [94]								●			●		●	
Diabetes-39 (D-39) [48, 50, 95–99]						●		●	●		●			●
Diabetes-39 scale (D-39) Short Form [100]						●		●	●		●			●
Diabetes Care Profile (DCP) [29, 30, 101]								●			●			●
Depressive Cognition Scale (DCS) [102, 103]							●							
Diabetes Diet-Related Quality of Life (DDRQOL) Scale [104]			●							●	●			●
Diabetes Diet-Related Quality of Life (DDRQOL)-R [105]														●
Diabetes Diet-Related Quality of Life (DDRQOL)-R Short Form [105]														●
Brief Diabetes Distress Screening (DDS)-2 [106]					●									
17-item Diabetes Distress Scale (DDS-17) [107–117]					●	●	●							
Diabetes Distress Scale (DDS)-Thai [118]					●	●	●							
Diabetes Distress Scale (DDS)-Saudi-Arabian [116]					●	●	●							
Depression in Diabetes Self-Rating Scale [119]							●							
Dreiser's Functional Hand Index (DFI) [120]								●						
Diabetes Foot Ulcer Scale (DFS) [31]					●	●	●	●			●			●
Diabetes Foot Ulcer Scale (DFS-SF) [121–125]					●	●	●	●						●
Diabetes Health Profile (DHP)-18 [126–130]					●	●								●
Diabetes Health Profile (DHP)-31 [131]					●	●								●
DAWN2 Impact of Diabetes Profile (DIDP)-6 [32]								●		●	●			●
DAWN2 Impact of Diabetes Profile (DIDP)-7 [32]								●		●	●			●
Diabetes Impact Measurement Scales (DIMS) [132, 133]	●										●		●	●
Diabetes-Specific Quality of Life Questionnaire (DMQoL) [134, 135]												●		
Diabetes Quality of Life Clinical Trial Questionnaire (DQLCTQ) [35]	●		●		●	●		●		●	●	●		●
Diabetes Quality of Life Clinical Trial Questionnaire-Revised (DQLCTQ-Rev) [35]	●		●		●			●		●				●
Asian Diabetes Quality of Life (DQOL)-Chinese-18 [33]			●							●	●			●
Asian Diabetes Quality of Life (DQOL)-English-21 [33, 136]			●							●	●			●
Asian Diabetes Quality of Life (DQOL)-Malay-21 [33]			●							●	●			●
Diabetes Quality of Life (DQOL-15) [70, 137–140]						●						●		●
Diabetes Quality of Life (DQOL)- Afaan Oromoo-34 [141]						●						●		●
Diabetes Quality of Life (DQOL)-42 [142–144]						●						●		●
Diabetes Quality of Life (DQOL)-45 [145]						●						●		●
Diabetes Quality of Life (DQOL)-46 [51, 146–148]						●						●		●
Diabetes Quality of Life (DQOL)-60 [149]						●						●		●
Diabetes Quality of Life (DQOL) revised version [150]						●						●		●
Diabetes Quality of Life (DQOL)-Brazil [151]						●						●		●
Diabetes Quality of Life (DQOL)-Brazil-8 [152]						●						●		●
Diabetes Quality of Life (DQOL)-Chinese-24 [153]						●						●		●
Diabetes Quality of Life (DQOL)-Arabic-29 [154]						●						●		●
Diabetes Quality of Life (DQOL)-Spanish-43 [155]						●						●		●
Iranian Diabetes Quality of Life (IRDQOL)-41 [156, 157]												●	●	
Diabetes-specific Quality of Life scale (D-QOL)-34 [158]	●				●	●	●				●			●
Type 2 Diabetes Symptom Checklist (DSC) [159]	●	●	●							●				
Diabetes Symptom Checklist-Revised (DSC-R) [160, 161]	●	●	●							●				
Korean- Diabetes Symptom Checklist-Revised (K-DSC-R) [162]	●	●	●											
Diabetes Symptom Self-Care Inventory (DSSCI) [26]	●													
Elderly Diabetes Burden Scale (EDBS) [36]	●					●					●			●
Edinburgh Depression Scale (EDS) [163]							●							
EuroQol (EQ)-5D-3L [47, 55, 72, 127, 164–175]		●				●	●	●						●
EuroQol (EQ)-5D-5L [170–173, 175–179]		●				●	●	●						●
13-item Fatigue subscale of the FACIT-F [180]			●											
General well-being schedule [181]			●		●		●							●
Golombok-Rust Inventory of Sexual Satisfaction (GRISS) [182]									●					
Hand Function Disability Scale (HFDS) [120]								●						
the Worry subscale from the Hypoglycemia Fear Survey (HFS-W) [183]						●								
Hypoglycemia Perspectives Questionnaire (HPQ) [184]					●	●								●
Health Status Questionnaire (HSQ) 2.0 [38]		●	●					●		●	●	●		
Health Utilities Index Mark 2 (HUI2) [40]	●	●			●	●	●	●	●	●				●
Health Utilities Index Mark 3 (HUI3) [40, 185, 186]	●	●			●	●	●	●		●				
Impact of Weight on Quality of Life-Lite (IWQOL-lite) [187]					●			●	●		●			●
Impact of Weight on Quality of Life-Lite-Clinical Trials (IWQOL-Lite-CT) [188, 189]								●		●	●			
Kessler-10 Psychological Distress scale (K10) [129]	●		●			●	●							●
LQD Quality of Life with Diabetes (LQD) [48]					●									●
Menopause-specific Quality of Life (MENQOL) [190]	●							●	●	●	●			
Michigan Hand Outcomes Questionnaire (MHQ) [120]		●						●			●			●
MOS Six-Item Sleep Scale [191]				●										
National Diabetes Register (NDR) survey [192–194]					●	●					●			●
Neuropathy- and Foot Ulcer–Specific Quality of Life instrument (NeuroQoL) [195]	●	●			●	●	●	●						
Nottingham Health Profile (NHP) [196]		●	●	●	●	●	●	●	●		●			
Short Form Problem Areas in Diabetes (PAID)-1 [197]					●	●	●							
Problem areas in diabetes (PAID)-5 [197–199]					●	●	●							
Problem areas in diabetes (PAID)-16 [200, 201]					●	●	●							●
Problem areas in diabetes (PAID)-20 [111, 112, 129, 201–216]					●	●	●							●
Psychological General Well-Being Index (PGWB) [169]			●			●	●					●	●	●
Patient-reported outcomes in Thai patients with type 2 diabetes mellitus (PRO-DM-Thai) [28]	●							●		●	●	●		●
Pittsburg Sleep Quality Index (PSQI) [217]				●										
Quality of life (QOL) questionnaire [37]					●	●							●	●
Impact of hypoglycemia on the HRQoL of type 2 diabetes patients (QoLHYPO^©^) questionnaire [218]												●		
Quality of Life for Indian diabetes Patients (QOLID) [34]	●							●		●	●	●		●
Quality of life scale for Iraqi Diabetes patients (QOLSID) [219]					●									●
Self-completion instrument for diabetes [220]	●				●		●	●						
Self-perception of health [39]					●			●			●	●		●
12-Item Short Form Health Survey (SF-12) [40–42]		●	●					●		●	●	●		
20-item Short Form Health Survey (SF-20) [43]		●						●		●	●	●		
36-Item Short Form Health Survey (SF-36) [29, 44–55]		●	●					●		●	●	●		
Short Form Problem Areas in Diabetes in Chinese (SF-PAID-C) [221]					●	●	●							●
Patient Health Questionnaire (PHQ)-4 [222]							●	●						
Patient Health Questionnaire (PHQ)-9 [223–226]							●							
Pictorial Representation of Illness and Self Measure Revised II (PRISM-RII) [27]												●	●	
Questionnaire on Stress in Diabetic Patients (QSD) [227]	●					●					●			●
Questionnaire on Stress in Patients with Diabetes-Revised (QSD-R) [228]	●						●	●	●		●			●
Questionnaire on Stress in Patients with Diabetes-Revised-Turkish (QSD-R-TR) [229]	●					●		●	●		●			●
Questionnaire on Stress in Patients with Diabetes-Revised-Turkish-Cyprus (QSD-R-TR) [230]					●	●	●				●			●
Sexual Quality of Life questionnaire-Female (SQOL-F) [231]									●					
Type 2 Diabetes Distress Assesment System (T2-DDAS) [232]		●				●	●							●
Well-being questionnaire (W-BQ) 12 [233, 234]			●							●				
Well-being questionnaire (W-BQ) 22 [24, 48, 235]			●			●	●			●			●	
Well-being questionnaire (W-BQ) 28 [236]			●		●					●				
Well-being and Treatment Satisfaction scales (W-BQ) [237]						●	●						●	
Well-being Enquiry for Diabetics (WED) [56]	●									●		●		●
The World Health Organisation- Five Well-Being Index (WHO-5) [55, 238–241]													●	
World Health Organisation Quality of Life (WHOQOL-100) [23]					●	●	●	●			●			●
World Health Organisation Quality of Life (WHOQOL)-BREF [24, 25, 44, 157, 242]					●	●	●	●			●			●

Within the symptom status level, 31/91 of the PROMs (subscales) measured physical symptoms, including pain, energy/fatigue and sleep as well as 69/91 measured mental symptoms, including distress, anxiety/worry and depression. With regard to the functional status level, 40/60 of the PROMs measured physical function, including activities of daily living and sexual function, 28/60 measured psychological function and 38/60 measured social/role function. There is a lot of heterogeneity, for example in the social function level, with many different constructs being measured, such as social well-being, restriction of social function, social role fulfillment and psychosocial disabilities, but also having friends, work and relationships, alienation, barriers and social burden.

In addition, 16/116 of the PROMs measured global quality of life. 61/116 of the HRQOL PROMs also include characteristics of the individual or environment and even PREMs, rather than only aspects of HRQOL. This includes characteristics of the individual, for example positive attitude [29–31], characteristics of the environment such as financial situation [31–34] or PREMs, such as treatment satisfaction [28, 34–37]. For one PROM it was specifically mentioned that demographics were also assessed as part of the PROM, namely the Diabetes Quality of Life Clinical Trial Questionnaire (DQLCTQ) [35].

Finally, only 9/116 of the HRQOL PROMs measured all aspects of HRQOL based on the Wilson & Cleary model. These PROMs include the DQLCTQ [35], Health Status Questionnaire (HSQ) 2.0 [38], PRO-DM-Thai [28], Quality of Life for Indian diabetes Patients (QOLID) [34], Self-perception of health [39], 12-Item Short Form Health Survey (SF-12) [40–42], 20-item Short Form Health Survey (SF-20) [43], 36-Item Short Form Health Survey (SF-36) [29, 44–55] and Well-being Enquiry for Diabetics (WED) [56]. Also, despite the fact that the authors of the included papers claimed that the PROM aims to measure at least (aspects of) symptom status, functional status, general health perceptions or HRQOL, 8/116 of the PROMs measured only global quality of life or PREMs and no HRQOL construct(s).

## Discussion

In our systematic review of the literature, from a total of 220 studies, we identified 116 unique PROMs aiming to measure (aspects of) HRQOL in people with type 2 diabetes. Of these HRQOL PROMs, 80% (of the subscales) measured symptom status, 50% measured functional status and 20% measured general health perceptions. In addition, 15% of the PROMs (subscales) measured global quality of life. 50% of the 116 PROMs (subscales) also include characteristics of the individual (e.g. aspects of personality, coping) or environment (e.g. social or financial support) and patient-reported experience measures (PREMs, e.g. measure of a patient's perception of their personal experience of the healthcare they have received, e.g. treatment satisfaction), which are not part of the HRQOL construct. The (sub-)scales of these PROMs thus presented a great heterogeneity of constructs, with about 5% of the PROMs measuring all aspects of HRQOL based on the Wilson & Cleary model and about 5% not measuring HRQOL (constructs) at all. This review shows the great amount of PROMs developed. Furthermore, some PROMs are very long, which may suggest poor acceptability.

When conducting this review we faced multiple challenges. First, the terminology used for the constructs the (subscales of the) PROMs measure was unclear and definitions of the constructs are mostly lacking. It was therefore unclear to us whether names of the PROMs and subscales represent different or the same concepts. This large variability in operationalization of HRQOL made it difficult to classify the PROMs. This lack of clarity about what a PROM actually measures also makes it difficult or even impossible to know whether a PROM has good validity (i.e. whether it measures what it is supposed to measure). A second challenge was that information regarding the characteristics of the PROMs was often lacking or misleading. For example, the availability and the number and names of (sub-)scales and the number of items per (sub-)scale were often not presented in the paper. As a result we had to consult additional resources, such as other articles, Google (e.g. manuals or websites) or the PROQOLID database [22]. However, even this strategy sometimes failed, which may have resulted in an incomplete overview of the PROMs (Table 1). This poor reporting is possibly due to older papers not meeting our modern day standards, but hampers researchers and health care providers to select the best PROM for their purpose. The poor information status and very large hetereogeneity in PROMs (subscales) is not unique to the diabetes field [243]. PROMs are increasingly used as primary outcome measures in studies and tools for clinical decision making. The poor state makes it very difficult, and potentially even impossible, to compare study results or cohorts directly, since all PROMs measure different constructs and thus different outcomes. In this review, we did not systematically evaluate the measurement properties of the PROMs, such as content validity, construct validity, reliability and responsiveness. Therefore, researchers should be careful when using this review to select PROMs as we cannot guarantee that the content of the PROMs or subscales really match the intended construct and we cannot guarantee that the PROMs are reliable and responsive to change [244].

This review highlights the great amount of PROMs developed and used and the heterogeneity of their content. We feel there is a need to reach consensus on which PROM to measure HRQOL as well as which HRQOL aspects are most important to measure for people with type 2 diabetes. One solution is the development of Core Outcome Sets (COS) or Standard Sets, which are agreed sets of outcomes (and associated measurement instruments) to be measured in all trials or clinical practice. International organizations such as COMET (https://www.comet-initiative.org/) and ICHOM (www.ichom.org) have developed such COSs for type 2 diabetes [245–247]. However, the value of these COSs are limited, because they have a strong focus on biological outcomes, such as glycemic control [199–201] and there was limited input from people with expertise in PRO measurement or people with type 2 diabetes. This resulted in dissimilar recommendations regarding PROMs between the initiatives, but also inclusion of the ‘Diabetes Treatment Satisfaction Questionnaire’ (which is a PREM) and only inclusion of activities of daily living and overall quality of life, and no other aspects of HRQOL [245–247]. Qualitative studies show the importance of ‘To live a good life with diabetes’ for people with type 2 diabetes [248].

## Limitations and strengths

This systematic review has several limitations and strengths. The first limitation is that the classification of the constructs was made based on reviewing the names of (sub)scales and not their content. We acknowledge that this may have resulted in misclassification, because of misleading construct names that do not reflect the content. It would have been better to look at the content of the PROMs to determine what aspects of HRQOL they measure, rather than using the names of the instrument (scales). We have done so for part of the PROMs, i.e. only the disease-specific HRQOL PROMs, in a separate review [244] where we did a full content validity assessment of these PROMs. However, the fact that there might be a mismatch between our classification and what the PROMs actually measure is a striking finding of this review. It is problematic that the name and description of a PROM as published in the literature does not tell us, or may even mislead us about what the PROM actually measures. This strongly hampers researchers and clinicians to select the optimal PROM for their purpose. Second, even though using an extensive search string, we identified 27% of the included studies from reference lists. However, by using this extensive search strategy our review is more complete than previous reviews specifically on HRQOL in those with type 2 diabetes. For example, we identified over 50 HRQOL PROMs with our search that were not found in the Wee et al. review [15]. We speculate this discrepancy is due to their lack of reference checking. Strengths of this systematic review were the extensive search with no restrictions on publication data or language as well as reference checking. Second, the use of a conceptual model to assess which aspects of HRQOL were measured by PROM (subscales) provides helpful information for researchers and health care providers searching for a PROM to measure one or more specific aspects of HRQOL, that is not provided in previous reviews. As stated before, instrument selection should be based on which relevant aspects of HRQOL one wants to measure and different aspects of HRQOL can be measured with subscales from different PROMs. Even though the Wilson and Cleary model is the most frequently used, other conceptual models are available that might be preferred by other researchers [4]. However, our conclusion on the heterogeneity and lack of clarity of constructs being measured with PROMs in the diabetes field would not have been different. Finally, despite our systematic review providing an overview and identifying the difficulties of the field, it also provides caution and food for thought regarding the use of the PROMs. Future studies are needed to provide definitive recommendations on which PROMs to use in people with type 2 diabetes.

## Conclusion

A large number of PROMs are available for people with type 2 diabetes, which intend to measure (aspects of) HRQOL. These PROMs measure a large variety of (sub)constructs, which are not all HRQOL constructs, with a small amount of PROMs not measuring HRQOL at all. There is a need for consensus on which aspects of HRQOL should be measured in people with type 2 diabetes and which PROMs to use in research and daily practice.

### Supplementary information

Below is the link to the electronic supplementary material.

Supplementary file1 (DOCX 40 KB)

## Appendix 1 Search strategy

### PubMed search April 29, 2019

#### #1 Diabetes type 2

((Diabet*[tiab] AND (("non insulin"[tiab] AND depend*[tiab]) OR ("noninsulin"[tiab] AND depend*[tiab]) OR “type 2”[tiab] OR “type II” [tiab])) OR iddm[tiab] OR niddm[tiab] OR “glucose intolerance”[tiab] OR “insulin resistant”[tiab] OR “insulin resistance”[tiab]).

#### #2 Modified filter for studies on measurement properties*

"Validation Studies"[pt] OR "psychometrics"[MeSH] OR psychometr*[tiab] OR clinimetr*[tw] OR clinometr*[tw] OR "outcome assessment (health care)"[MeSH] OR "outcome assessment"[tiab] OR "outcome measure*"[tw] OR "observer variation"[MeSH] OR "observer variation"[tiab] OR "reproducibility of results"[MeSH] OR reproducib*[tiab] OR "discriminant analysis"[MeSH] OR reliab*[tiab] OR unreliab*[tiab] OR valid*[tiab] OR "coefficient of variation"[tiab] OR homogeneity[tiab] OR homogeneous[tiab] OR "internal consistency"[tiab] OR (cronbach*[tiab] AND (alpha[tiab] OR alphas[tiab])) OR (item[tiab] AND (correlation*[tiab] OR selection*[tiab] OR reduction*[tiab])) OR agreement[tw] OR precision[tw] OR imprecision[tw] OR "precise values"[tw] OR test–retest[tiab] OR (test[tiab] AND retest[tiab]) OR (reliab*[tiab] AND (test[tiab] OR retest[tiab])) OR stability[tiab] OR interrater[tiab] OR inter-rater[tiab] OR intrarater[tiab] OR intra-rater[tiab] OR intertester[tiab] OR inter-tester[tiab] OR intratester[tiab] OR intra-tester[tiab] OR interobserver[tiab] OR inter-observer[tiab] OR intraobserver[tiab] OR intra-observer[tiab] OR intertechnician[tiab] OR inter-technician[tiab] OR intratechnician[tiab] OR intra-technician[tiab] OR interexaminer[tiab] OR inter-examiner[tiab] OR intraexaminer[tiab] OR intra-examiner[tiab] OR interassay[tiab] OR inter-assay[tiab] OR intraassay[tiab] OR intra-assay[tiab] OR interindividual[tiab] OR inter-individual[tiab] OR intraindividual[tiab] OR intra-individual[tiab] OR interparticipant[tiab] OR inter-participant[tiab] OR intraparticipant[tiab] OR intra-participant[tiab] OR kappa[tiab] OR kappa's[tiab] OR kappas[tiab] OR repeatab*[tw] OR ((replicab*[tw] OR repeated[tw]) AND (measure[tw] OR measures[tw] OR findings[tw] OR test[tw] OR tests[tw])) OR generaliza*[tiab] OR generalisa*[tiab] OR concordance[tiab] OR (intraclass[tiab] AND correlation*[tiab]) OR discriminative[tiab] OR "known group"[tiab] OR "factor analysis"[tiab] OR "factor analyses"[tiab] OR "factor structure"[tiab] OR "factor structures"[tiab] OR subscale*[tiab] OR (multitrait[tiab] AND scaling[tiab] AND (analysis[tiab] OR analyses[tiab])) OR "item discriminant"[tiab] OR "interscale correlation*"[tiab] OR error[tiab] OR errors[tiab] OR "individual variability"[tiab] OR "interval variability"[tiab] OR "rate variability"[tiab] OR (variability[tiab] AND (analysis[tiab] OR values[tiab])) OR "standard error of measurement"[tiab] OR responsive*[tiab] OR (limit[tiab] AND detection[tiab]) OR "minimal detectable concentration"[tiab] OR interpretab*[tiab] OR ((minimal[tiab] OR minimally[tiab] OR clinical[tiab] OR clinically[tiab]) AND (important[tiab] OR detectable[tiab]) AND (change[tiab] OR difference[tiab])) OR (small*[tiab] AND (real[tiab] OR detectable[tiab]) AND (change[tiab] OR difference[tiab])) OR "meaningful change"[tiab] OR "ceiling effect"[tiab] OR "floor effect"[tiab] OR "Item response model"[tiab] OR IRT[tiab] OR Rasch[tiab] OR "Differential item functioning"[tiab] OR DIF[tiab] OR "computer adaptive testing"[tiab] OR "item bank"[tiab] OR "cross-cultural equivalence"[tiab].

#### #3 PROM filter (developed by the University of Oxford, see www.comin.nl)

(HR-PRO[tiab] OR HRPRO[tiab] OR HRQL[tiab] OR HRQoL[tiab] OR QL[tiab] OR QoL[tiab] OR quality of life[tw] OR life quality[tw] OR health index*[tiab] OR health indices[tiab] OR health profile*[tiab] OR health status[tw] OR ((patient[tiab] OR self[tiab] OR child[tiab] OR parent[tiab] OR carer[tiab] OR proxy[tiab]) AND ((report[tiab] OR reported[tiab] OR reporting[tiab]) OR (rated[tiab] OR rating[tiab] OR ratings[tiab]) OR based[tiab] OR (assessed[tiab] OR assessment[tiab] OR assessments[tiab]))) OR ((disability[tiab] OR function[tiab] OR functional[tiab] OR functions[tiab] OR subjective[tiab] OR utility[tiab] OR utilities[tiab] OR wellbeing[tiab] OR well being[tiab]) AND (index[tiab] OR indices[tiab] OR instrument[tiab] OR instruments[tiab] OR measure[tiab] OR measures[tiab] OR questionnaire[tiab] OR questionnaires[tiab] OR profile[tiab] OR profiles[tiab] OR scale[tiab] OR scales[tiab] OR score[tiab] OR scores[tiab] OR status[tiab] OR survey[tiab] OR surveys[tiab]))).

(#1 AND #2 AND #3) NOT ("addresses"[Publication Type] OR "biography"[Publication Type] OR "case reports"[Publication Type] OR "comment"[Publication Type] OR "directory"[Publication Type] OR "editorial"[Publication Type] OR "festschrift"[Publication Type] OR "interview"[Publication Type] OR "lectures"[Publication Type] OR "legal cases"[Publication Type] OR "legislation"[Publication Type] OR "letter"[Publication Type] OR "news"[Publication Type] OR "newspaper article"[Publication Type] OR "patient education handout"[Publication Type] OR "popular works"[Publication Type] OR "congresses"[Publication Type] OR "consensus development conference"[Publication Type] OR "consensus development conference, nih"[Publication Type] OR "practice guideline"[Publication Type]) NOT ("animals"[MeSH Terms] NOT "humans"[MeSH Terms]).

### EMBASE search April 29, 2019

#### #1 Diabetes type 2

(Diabet*:ti,ab AND (('non insulin':ti,ab AND depend*:ti,ab) OR (noninsulin:ti,ab AND depend*:ti,ab) OR 'type 2':ti,ab OR 'type II':ti,ab)) OR iddm:ti,ab OR niddm:ti,ab OR 'glucose intolerance':ti,ab OR 'insulin resistant':ti,ab OR 'insulin resistance':ti,ab.

#### #2 Modified filter for studies on measurement properties*

'data collection method'/exp OR 'validation study'/exp OR 'feasibility study'/exp OR 'pilot study'/exp OR 'psychometry'/exp OR 'reproducibility'/exp OR reproducib*:ab,ti OR 'audit':ab,ti OR psychometr*:ab,ti OR clinimetr*:ab,ti OR clinometr*:ab,ti OR 'observer variation'/exp OR 'observer variation':ab,ti OR 'discriminant analysis'/exp OR 'validity'/exp OR reliab*:ab,ti OR valid*:ab,ti OR 'internal consistency':ab,ti OR (cronbach*:ab,ti AND ('alpha':ab,ti OR 'alphas':ab,ti)) OR 'item correlation':ab,ti OR 'item correlations':ab,ti OR 'item selection':ab,ti OR 'item selections':ab,ti OR 'item reduction':ab,ti OR 'item reductions':ab,ti OR 'agreement':ab,ti OR 'precision':ab,ti OR 'imprecision':ab,ti OR 'precise values':ab,ti OR 'test–retest':ab,ti OR ('test':ab,ti AND 'retest':ab,ti) OR (reliab*:ab,ti AND ('test':ab,ti OR 'retest':ab,ti)) OR 'stability':ab,ti OR 'interrater':ab,ti OR 'inter-rater':ab,ti OR 'intrarater':ab,ti OR 'intra-rater':ab,ti OR 'intertester':ab,ti OR 'inter-tester':ab,ti OR 'intratester':ab,ti OR 'intra-tester':ab,ti OR 'interobeserver':ab,ti OR 'inter-observer':ab,ti OR 'intraobserver':ab,ti OR 'intra-observer':ab,ti OR 'intertechnician':ab,ti OR 'inter-technician':ab,ti OR 'intratechnician':ab,ti OR 'intra-technician':ab,ti OR 'interexaminer':ab,ti OR 'inter-examiner':ab,ti OR 'intraexaminer':ab,ti OR 'intra-examiner':ab,ti OR 'interassay':ab,ti OR 'inter-assay':ab,ti OR 'intraassay':ab,ti OR 'intra-assay':ab,ti OR 'interindividual':ab,ti OR 'inter-individual':ab,ti OR 'intraindividual':ab,ti OR 'intra-individual':ab,ti OR 'interparticipant':ab,ti OR 'inter-participant':ab,ti OR 'intraparticipant':ab,ti OR 'intra-participant':ab,ti OR 'kappa':ab,ti OR 'kappas':ab,ti OR 'coefficient of variation':ab,ti OR repeatab*:ab,ti OR (replicab*:ab,ti OR 'repeated':ab,ti AND ('measure':ab,ti OR 'measures':ab,ti OR 'findings':ab,ti OR 'test':ab,ti OR 'tests':ab,ti)) OR generaliza*:ab,ti OR generalisa*:ab,ti OR 'concordance':ab,ti OR ('intraclass':ab,ti AND correlation*:ab,ti) OR 'discriminative':ab,ti OR 'known group':ab,ti OR 'factor analysis':ab,ti OR 'factor analyses':ab,ti OR 'factor structure':ab,ti OR 'factor structures':ab,ti OR 'dimensionality':ab,ti OR subscale*:ab,ti OR 'multitrait scaling analysis':ab,ti OR 'multitrait scaling analyses':ab,ti OR 'item discriminant':ab,ti OR 'interscale correlation':ab,ti OR 'interscale correlations':ab,ti OR ('error':ab,ti OR 'errors':ab,ti AND (measure*:ab,ti OR correlat*:ab,ti OR evaluat*:ab,ti OR 'accuracy':ab,ti OR 'accurate':ab,ti OR 'precision':ab,ti OR 'mean':ab,ti)) OR 'individual variability':ab,ti OR 'interval variability':ab,ti OR 'rate variability':ab,ti OR 'variability analysis':ab,ti OR 'standard error of measurement':ab,ti OR responsive*:ab,ti OR ('limit':ab,ti AND 'detection':ab,ti) OR 'minimal detectable concentration':ab,ti OR interpretab*:ab,ti OR (small*:ab,ti AND ('real':ab,ti OR 'detectable':ab,ti) AND ('change':ab,ti OR 'difference':ab,ti)) OR 'meaningful change':ab,ti OR 'minimal important change':ab,ti OR 'minimal important difference':ab,ti OR 'minimally important change':ab,ti OR 'minimally important difference':ab,ti OR 'minimal detectable change':ab,ti OR 'minimal detectable difference':ab,ti OR 'minimally detectable change':ab,ti OR 'minimally detectable difference':ab,ti OR 'minimal real change':ab,ti OR 'minimal real difference':ab,ti OR 'minimally real change':ab,ti OR 'minimally real difference':ab,ti OR 'ceiling effect':ab,ti OR 'floor effect':ab,ti OR 'item response model':ab,ti OR 'irt':ab,ti OR 'rasch':ab,ti OR 'differential item functioning':ab,ti OR 'dif':ab,ti OR 'computer adaptive testing':ab,ti OR 'item bank':ab,ti OR 'cross-cultural equivalence':ab,ti.

#### #3 PROM filter (developed by the University of Oxford, see www.comin.nl)

(HR-PRO:ti,ab OR HRPRO:ti,ab OR HRQL:ti,ab OR HRQoL:ti,ab OR QL:ti,ab OR QoL:ti,ab OR 'quality of life':ti,ab OR 'life quality':ti,ab OR 'health index*':ti,ab OR 'health indices':ti,ab OR 'health profile*':ti,ab OR 'health status':ti,ab OR ((patient:ti,ab OR self:ti,ab OR child:ti,ab OR parent:ti,ab OR carer:ti,ab OR proxy:ti,ab) AND ((report:ti,ab OR reported:ti,ab OR reporting:ti,ab) OR (rated:ti,ab OR rating:ti,ab OR ratings:ti,ab) OR based:ti,ab OR (assessed:ti,ab OR assessment:ti,ab OR assessments:ti,ab))) OR ((disability:ti,ab OR function:ti,ab OR functional:ti,ab OR functions:ti,ab OR subjective:ti,ab OR utility:ti,ab OR utilities:ti,ab OR wellbeing:ti,ab OR 'well being':ti,ab) AND (index:ti,ab OR indices:ti,ab OR instrument:ti,ab OR instruments:ti,ab OR measure:ti,ab OR measures:ti,ab OR questionnaire:ti,ab OR questionnaires:ti,ab OR profile:ti,ab OR profiles:ti,ab OR scale:ti,ab OR scales:ti,ab OR score:ti,ab OR scores:ti,ab OR status:ti,ab OR survey:ti,ab OR surveys:ti,ab))).

#### #4 publicatie types

#3 AND ('article'/it OR 'article in press'/it OR 'review'/it).

#### #5 not animals

#4 NOT ([animals]/lim NOT [humans]/lim).

* Modified from Terwee et al. (19): a few search terms were left out to decrease the number of abstracts needed to be read.

## Abbreviations

COS: Core Outcome Sets
HRQOL: Health-Related Quality Of Life
PRO: Patient Reported Outcome
PROM: Patient Reported Outcome Measure
PRISMA: Preferred Reporting Items for Systematic Reviews and Meta-Analysis
PREM: Patient-Reported Experience Measures
QOL: Quality Of Life
