# Supplementary material for: Patient-reported outcome measures for assessing health-related quality of life in people with type 2 diabetes: A systematic review
Source: Rev Endocr Metab Disord. 2022 Jul 2;23(5):931–77. doi: 10.1007/s11154-022-09734-9 (PMC9515038; doi:10.1007/s11154-022-09734-9)
Supplement: Supplementary file 1 — Supplementary file1 (DOCX 40 KB) [file 11154_2022_9734_MOESM1_ESM.docx]

| **Supplemental table 1.**  Overview of the specific levels of HRQOL that the included PROMs measure based on the Wilson and Cleary model (5) | | | | | | | | | | | | | | |
| --- | --- | --- | --- | --- | --- | --- | --- | --- | --- | --- | --- | --- | --- | --- |
|  | | | | | | | | | | | | | | |
| **PROM** | **Health-related quality of life** | | | | | | |  | | | |  | **Other** |  |
|  | **Symptom status** | | | | | | | **Functional status** | | | | **General health perception** | **Global quality of life**  **and life satisfaction** | **Characteristics of**  **individual/environment or PREM** |
|  | **Disease specific symptoms** | **Physical symptoms** | | | **Mental symptoms** | | | **Physical**  **function** | | **Psychological function** | **Social function** | **Overall health** | **Overall quality of life** |  |
|  |  | **Pain** | **Energy/**  **fatigue** | **Sleep** | **Distress** | **Anxiety/worry** | **Depression** | **Activities of daily living** | **Sexual function** | **Emotional function/ cognition** | **Social function/ participation** | **General health perceptions, self-rated health** | **Overall quality of life/well-being** |  |
| 15D standardized measure of health-related quality of life Finnish (15D Finnish) | Breathing  Speech  Vision  Hearing | Discomfort and its symptoms | Vitality | Sleeping | Distress |  | Depression | Mobility  Usual activities  Eating | Sexual activity | Mental function |  |  |  | Elimination |
| Audit of Diabetes Dependent Quality of Life (ADDQOL)-13 |  |  |  |  |  |  |  |  |  |  |  |  | Quality of life |  |
| Audit of Diabetes Dependent Quality of life (ADDQOL)-16 |  |  |  |  |  |  |  |  |  |  |  |  | Quality of life |  |
| Audit of Diabetes Dependent Quality of life (ADDQOL) 17-senior |  |  |  |  |  |  |  |  |  |  |  |  | Quality of life |  |
| Audit of Diabetes Dependent Quality of Life (ADDQOL)-18 |  |  |  |  |  |  |  |  |  |  |  |  | Quality of life |  |
| Audit of Diabetes Dependent Quality of Life (ADDQOL)- 19 |  |  |  |  |  |  |  |  |  |  |  |  | Quality of life |  |
| A Health status instrument developed for South-African women |  |  |  |  |  |  |  |  |  | Mental well-being | Social well-being |  |  |  |
| The Ability to Perform Physical Activities of Daily Living Questionnaire (APPADL) |  |  |  |  |  |  |  | Ability to perform daily physical activities |  |  |  |  |  |  |
| Attitudes to Diabetes (ATT)-19 |  |  |  |  |  |  |  |  |  | Psychological adjustment to diabetes |  |  |  |  |
| Attitude to Diabetes (ATT)-39 |  |  |  |  | Stress |  |  |  |  |  | Alienation |  |  | Adaptation  Guilt  Illness conviction  Tolerance for ambiguity |
| Chinese Diabetes Distress screening (CDDS)-15 |  |  |  |  | Emotional burden  Regimen- and social support related distress  Physician-related distress | Emotional burden | Emotional burden |  |  |  |  |  |  |  |
| Centre for Epidemiological Studies Depression Scale (CESD) |  |  |  |  |  |  | Depressed affect  Somatic-retarded activity  Interpersonal affect  Positive affect |  |  |  |  |  |  |  |
| Clinically Useful Depression Outcome Scale (CUDOS) |  |  |  |  |  |  | Symptoms of depression |  |  | Psychosocial disabilities | Psychosocial disabilities |  | Effect of depression on quality of life |  |
| Diabetes-39 (D-39) |  |  |  |  |  | Anxiety and worry |  | Energy and mobility | Sexual functioning |  | Social burden |  |  | Diabetes control |
| Diabetes-39 scale (D-39) Short Form |  |  |  |  |  | Anxiety and worry |  | Energy and mobility | Sexual functioning |  | Social burden |  |  | Diabetes control |
| Diabetes Care Profile (DCP) |  |  |  |  |  |  |  | Exercise barriers |  |  | Social and personal factors |  |  | Control problems  Positive attitude  Negative attitude  Self-care ability  Importance of care  Self-care adherence  Diet adherence  Medical barriers  Monitoring barriers  Understanding management practice  Long-term care benefits  Support attitudes |
| Depressive Cognition Scale (DCS) |  |  |  |  |  |  | Depressive cognition |  |  |  |  |  |  |  |
| Diabetes Diet-Related Quality of Life (DDRQOL) Scale |  |  | Vitality |  |  |  |  |  |  | Mental health | Restriction of social functions |  |  | Satisfaction with diet  Burden of diet therapy  Perceived merits of diet therapy  General perception of diet |
| Diabetes Diet-Related Quality of Life (DDRQOL)-R |  |  |  |  |  |  |  |  |  |  |  |  |  | Satisfaction with diet  Burden of diet therapy  Perceived merits of diet therapy |
| Diabetes Diet-Related Quality of Life (DDRQOL)-R Short Form |  |  |  |  |  |  |  |  |  |  |  |  |  | Satisfaction with diet  Burden of diet therapy  Perceived merits of diet therapy |
| Brief Diabetes Distress Screening (DDS)-2 |  |  |  |  | Diabetes distress |  |  |  |  |  |  |  |  |  |
| 17-item Diabetes Distress Scale (DDS-17) |  |  |  |  | Emotional burden  Physician-related distress  Regimen-related distress  Interpersonal distress | Emotional burden | Emotional burden |  |  |  |  |  |  |  |
| Diabetes Distress Scale (DDS)-Thai |  |  |  |  | Emotional and regimen-related burden  Physician- and nurse-related distress  Regimen-related distress  Interpersonal distress | Emotional and regimen-related burden | Emotional and regimen-related burden |  |  |  |  |  |  |  |
| Depression in Diabetes Self-Rating Scale |  |  |  |  |  |  | Depressive symptoms |  |  |  |  |  |  |  |
| Dreiser's Functional Hand Index (DFI) |  |  |  |  |  |  |  | Difficulties of 10 different daily activities to execute |  |  |  |  |  |  |
| Diabetes Foot Ulcer Scale (DFS) |  |  |  |  | Emotions | Emotions | Emotions | Leisure  Physical health  Daily activities |  |  | Family  Friends |  |  | Non-compliance  Positive attitude  Treatment  Satisfaction  Financial |
| Diabetes Foot Ulcer Scale (DFS-SF) |  |  |  |  | Negative emotions | Negative emotions  Worried about ulcers/feet | Negative emotions | Leisure  Physical health  Dependence/daily life |  |  |  |  |  | Bothered by ulcer care |
| Diabetes Health Profile (DHP)-18 |  |  |  |  | Psychological distress | Barriers to activity |  |  |  |  |  |  |  | Disinhibited eating |
| Diabetes Health Profile (DHP)-31 |  |  |  |  | Psychological distress | Barriers to activity |  |  |  |  |  |  |  | Disinhibited eating |
| DAWN2 Impact of Diabetes Profile (DIDP)-6 |  |  |  |  |  |  |  | Physical health  Leisure activities |  | Emotional wellbeing | Relationships  Work or studies |  |  | Financial situation |
| DAWN2 Impact of Diabetes Profile (DIDP)-7 |  |  |  |  |  |  |  | Physical health  Leisure activities |  | Emotional wellbeing | Relationships  Work or studies |  |  | Financial situation  Dietary freedom |
| Diabetes Impact Measurement Scales (DIMS) | Symptoms |  |  |  |  |  |  |  |  |  | Social role fulfillment |  | Well-being | Diabetes-related morale |
| Diabetes-Specific  Quality of Life Questionnaire (DMQoL) |  |  |  |  |  |  |  |  |  |  |  | Health-related quality of life |  |  |
| Diabetes Quality of Life Clinical Trial Questionnaire (DQLCTQ) | Frequency of symptoms  Bothersomeness of symptoms |  | Energy/ fatigue |  | Health distress | DQOL Social worry  DQOL Diabetes worry  Worry |  | Physical functioning |  | Mental health | Global role functioning  Social functioning  General social functioning | General health  Comparative health  DQOL Impact |  | DQOL Satisfaction  Treatment satisfaction  Treatment flexibility  Social stigma  Self-efficacy  Demographics |
| Diabetes Quality of Life Clinical Trial Questionnaire-Revised (DQLCTQ-Rev) | Frequency of symptoms |  | Energy/ fatigue |  | Health distress |  |  | Physical function |  | Mental health |  |  |  | Satisfaction  Treatment satisfaction  Treatment flexibility |
| Asian Diabetes Quality of Life (DQOL)-Chinese-18 |  |  | Energy levels |  |  |  |  |  |  | Memory | Relationship |  |  | Financial concerns  Diet and activities |
| Asian Diabetes Quality of Life (DQOL)-English-21 |  |  | Energy levels |  |  |  |  |  |  | Memory and cognition | Relationship |  |  | Financial  Diet |
| Asian Diabetes Quality of Life (DQOL)-Malay-21 |  |  | Energy levels |  |  |  |  |  |  | Memory and cognition | Relationship |  |  | Financial  Diet |
| Diabetes Quality of Life (DQOL-15) |  |  |  |  |  | Social/ worry  Vocational/worry |  |  |  |  |  | Impact |  | Satisfaction |
| Diabetes Quality of Life (DQOL) revised version |  |  |  |  |  | Worry |  |  |  |  |  | Impact |  | Satisfaction |
| Diabetes Quality of Life (DQOL)-42 |  |  |  |  |  | Diabetes-related worry |  |  |  |  |  | Impact |  | Satisfaction |
| Diabetes Quality of Life (DQOL)-45 |  |  |  |  |  | Diabetes-related worry  Social/vocational worry |  |  |  |  |  | Impact |  | Satisfaction |
| Diabetes Quality of Life (DQOL)-46 |  |  |  |  |  | Diabetes-related worry  Social/vocational worry |  |  |  |  |  | Impact |  | Satisfaction |
| Diabetes Quality of Life (DQOL)-60 |  |  |  |  |  | Disease related worries scale |  |  |  |  |  | Disease impact scale  General health questionnaire |  | Diabetes life satisfaction scale |
| Diabetes Quality of Life (DQOL)-Brazil |  |  |  |  |  | Concern: social/vocational  Concern: related to diabetes |  |  |  |  |  | Impact |  | Satisfaction |
| Diabetes Quality of Life (DQOL)-Brazil-8  ( |  |  |  |  |  | Concern: social/vocational  Concern: related to diabetes |  |  |  |  |  | Impact |  | Satisfaction |
| Diabetes Quality of Life (DQOL)-Chinese-24 |  |  |  |  |  | Worry |  |  |  |  |  | Impact |  | Satisfaction |
| Diabetes Quality of Life (DQOL)-Spanish-43 |  |  |  |  |  | Concern: social/vocational  Concern: related to diabetes |  |  |  |  |  | Impact |  | Satisfaction |
| Iranian Diabetes Quality of Life (IRDQOL)-41 |  |  |  |  |  |  |  |  |  |  |  | Health-related quality of life | General quality of life |  |
| Diabetes-specific Quality of Life scale (D-QOL)-34 | Diabetes- specific items |  |  |  | Emotional suffering | Emotional suffering | Emotional suffering |  |  |  | Social functioning |  |  | Adherence to treatment |
| Type 2 Diabetes Symptom Checklist (DSC) | Neuropathic sensoric  Cardiovascular  Vision  Hypoglycemic  Hyperglycemic | Neuropathic pain | Psychological fatigue |  |  |  |  |  |  | Psychological cognitive |  |  |  |  |
| Diabetes Symptom Checklist-Revised (DSC-R) | Neuropathic sensoric  Cardiovascular  Opthalmologic  Hypoglycemic  Hyperglycemic | Neuropathic pain | Psychological fatigue |  |  |  |  |  |  | Psychological cognitive |  |  |  |  |
| Korean- Diabetes Symptom Checklist- Revised (K-DSC-R) | Hypoglycemic  Ophthalmologic  Hyperglycemic  Cardiovascular  Sensory neuropathic | Neuropathic pain | Psychological fatigue |  |  |  |  |  |  |  |  |  |  |  |
| Diabetes Symptom Self-Care Inventory (DSSCI) | Diabetes symptoms |  |  |  |  |  |  |  |  |  |  |  |  |  |
| Elderly Diabetes Burden Scale (EDBS) | Symptom burden |  |  |  |  | Worry about diabetes |  |  |  |  | Social burden |  |  | Dietary restrictions  Treatment (dis)-satisfaction  Burden by tablets or insulin |
| Edinburgh Depression Scale (EDS) |  |  |  |  |  |  | Depression |  |  |  |  |  |  |  |
| EuroQol (EQ)-5D-3L |  | Pain/ discomfort |  |  |  | Anxiety/ depression | Anxiety/ depression | Mobility  Usual activities |  |  |  |  |  | Self-care |
| EuroQol (EQ)-5D-5L |  | Pain/ discomfort |  |  |  | Anxiety/ depression | Anxiety/ depression | Mobility  Usual activities |  |  |  |  |  | Self-care |
| 13-item Fatigue subscale of the FACIT-F |  |  | Fatigue |  |  |  |  |  |  |  |  |  |  |  |
| General well-being schedule |  |  | Vitality |  | Health concerns |  | Depression |  |  |  |  |  |  | Self-esteem |
| Golombok-Rust Inventory of Sexual Satisfaction (GRISS) |  |  |  |  |  |  |  |  | **Male**  Impotence  Premature ejaculation  Nonsensuality  Avoidance  Dissatisfaction  Infrequency  Noncommunication  **Female**  Vaginismu  Anorgasmia  Nonsensuality  Avoidance  Dissatisfaction  Infrequency  Noncommunication |  |  |  |  |  |
| Hand Function Disability Scale (HFDS) |  |  |  |  |  |  |  | Kitchen  Dressing  Hygiene  Office  Other |  |  |  |  |  |  |
| the Worry subscale from the Hypoglycemia Fear Survey (HFS-W) |  |  |  |  |  | Fear of hypoglycemia (worry subscale) |  |  |  |  |  |  |  |  |
| Hypoglycemia Perspectives Questionnaire (HPQ) |  |  |  |  | Symptom concern | Worry |  |  |  |  |  |  |  | Compensatory behavior |
| Health Status Questionnaire (HSQ) 2.0 |  | Bodily pain | Energy/ fatigue |  |  |  |  | Physical functioning |  | Mental health | Role limitations (due to physical health and/or emotional problems)  Social functioning | Health perception |  |  |
| Health Utilities Index Mark 2 (HUI2) | Sensation (hearing, vision and speech) | Pain |  |  | Emotion | Emotion | Emotion | Mobility | Fertility | Cognition |  |  |  | Self-care |
| Health Utilities Index Mark 3 (HUI3) | Vision  Hearing  Speech | Pain/ discomfort |  |  | Emotion | Emotion | Emotion | Ambulation  Dexterity |  | Cognition |  |  |  |  |
| Impact of Weight on Quality of Life-Lite (IWQOL-lite) |  |  |  |  | Public distress |  |  | Physical function | Sexual life |  | Work |  |  | Self-esteem |
| Impact of Weight on Quality of Life-Lite-Clinical Trials (IWQOL-Lite-CT) |  |  |  |  |  |  |  | Physical |  | Psychosocial | Psychosocial |  |  |  |
| Kessler-10 Psychological Distress scale (K10) | Motor agitation |  | Fatigue |  |  | Anxiety | Depressed mood |  |  |  |  |  |  | Worthless guilt |
| LQD Quality of Life with Diabetes (LQD) |  |  |  |  | Diabetes stress  Blood glucose stress |  |  |  |  |  |  |  |  | Diabetes satisfaction |
| Menopause-specific Quality of Life (MENQOL) | Vasomotor |  |  |  |  |  |  | Physical | Sexual functioning | Psychosocial | Psychosocial |  |  |  |
| Michigan Hand Outcomes Questionnaire (MHQ) |  | Pain |  |  |  |  |  | Overall hand function  Activities of daily living |  |  | Work performance |  |  | Aesthetics  Satisfaction with hand function |
| MOS Six-Item Sleep Scale |  |  |  | Initiation  Maintenance  Adequacy  Somnolence  Respiratory impairments |  |  |  |  |  |  |  |  |  |  |
| National Diabetes Register (NDR) survey |  |  |  |  | How you feel | Your worries |  |  |  |  | Barriers |  |  | Your capabilities to manage your diabetes  Support from others  Support from diabetes care providers  Medical devices and medical treatment |
| Neuropathy- and Foot Ulcer–Specific Quality of Life instrument (NeuroQoL) | Reduced feeling  Diffuse sensory motor | Pain |  |  | Interpersonal/emotional burden | Interpersonal/emotional burden | Interpersonal/emotional burden | Activity limitations |  |  |  |  |  |  |
| Nottingham Health Profile (NHP) |  | Pain | Energy | Sleep | Emotional reactions | Emotional reactions | Emotional reactions | Physical mobility  Jobs around the house  Home life  Hobbies  Holidays | Sex life |  | Social isolation  Paid employment  Social life |  |  |  |
| Short Form Problem Areas in Diabetes (PAID)-1 |  |  |  |  | Emotional problems | Emotional problems | Emotional problems |  |  |  |  |  |  |  |
| Problem areas in diabetes (PAID)-5 (162-164) |  |  |  |  | Emotional problems | Emotional problems | Emotional problems |  |  |  |  |  |  |  |
| Problem areas in diabetes (PAID)-16 |  |  |  |  | Emotional and management problems | Emotional and management problems | Emotional and management problems |  |  |  |  |  |  | Diabetes problems  Support problems |
| Problem areas in diabetes (PAID)-20 |  |  |  |  | Emotional problems/distress | Emotional problems/distress | Emotional problems/distress |  |  |  |  |  |  | Treatment problems  Food-related problems  Social support problems |
| Psychological General Well-Being Index (PGWB) |  |  | Vitality |  |  | Anxiety | Depressed mood |  |  |  |  | General health | Positive well-being | Self-control |
| Patient-reported outcomes in Thai patients with type 2 diabetes mellitus (PRO-DM-Thai) | Symptoms |  |  |  |  |  |  | Physical function |  | Psychological well-being | Social well-being | Global judgments of health |  | Self-care management  Satisfaction with care and flexibility of treatment |
| Pittsburg Sleep Quality Index (PSQI) |  |  |  | Subjective sleep quality  Sleep latency  Sleep duration  Habitual sleep efficiency  Sleep disturbances  Use of sleep medication  Daytime dysfunction |  |  |  |  |  |  |  |  |  |  |
| Quality of life (QOL) questionnaire |  |  |  |  | Degree of distress | Degree of apprehension |  |  |  |  |  |  | Degree of satisfaction with life | Degree of satisfaction with treatments |
| Impact of hypoglycemia on the HRQoL of T2DM patients (QoLHYPO©) questionnaire |  |  |  |  |  |  |  |  |  |  |  | Health-related quality of life |  |  |
| Quality of Life for Indian diabetes Patients (QOLID) | Symptom botherness |  |  |  |  |  |  | Physical endurance |  | Emotional/mental health | Social life, work and travel | General health |  | Treatment satisfaction  Financial worries  Diet advice tolerance |
| Self-completion instrument for diabetes | Symptoms |  |  |  | Psychological distress and barriers to activity |  | Depression | Physical function and energy |  |  |  |  |  |  |
| Self-perception of health |  |  |  |  | Worry about health |  |  | Dependence |  |  | Sociality | Feel healthy |  | Positive self-feeling  Attention seeking |
| 12-Item Short Form Health Survey (SF-12) |  | Pain | Vitality |  |  |  |  | Physical functioning  Role physical |  | Role emotional  Mental health | Social functioning | General health |  |  |
| 20-item Short Form Health Survey (SF-20) |  | Bodily pain |  |  |  |  |  | Physical functioning |  | Mental health | Role functioning  Social functioning | General health |  |  |
| 36-Item Short Form Health Survey (SF-36) |  | Pain | Energy and vitality |  |  |  |  | Physical functioning |  | Mental health | Social functioning  Role limitations due to physical problems  Role limitation due to emotional problems | General perception of health  Perceived change in health |  |  |
| Short Form Problem Areas in Diabetes in chinese (SF-PAID-C) |  |  |  |  | Emotional distress  Diabetes-related emotional problems | Diabetes-related emotional problems | Diabetes-related emotional problems |  |  |  |  |  |  | Problems related to food |
| Patient Health Questionnaire (PHQ)-9 |  |  |  |  |  |  | Depressive symptoms / depression |  |  |  |  |  |  |  |
| Pictorial Representation of Illness and Self Measure Revised II (PRISM-RII) |  |  |  |  |  |  |  |  |  |  |  | Illness perception measure | Self Illness separation (SIS) |  |
| Questionnaire on Stress in Diabetic Patients (QSD) | Problems with hypoglycemia |  |  |  |  | Fear of long-term complications |  |  |  |  | Problems with work  Strained doctor-patient relationship  Problems with relationship or family |  |  | Dietary restrictions  Difficulties with treatment regimen  Problems with acceptance  Reduction of performance  Feeling patronized |
| Questionnaire on Stress in Patients with Diabetes-Revised (QSD-R) | Hypoglycemia |  |  |  |  |  | Depression/fear of future | Physical complaints | Leisure time |  | Work  Partner  Doctor-patient relationship |  |  | Treatment regimen/diet |
| Questionnaire on Stress in Patients with Diabetes-Revised-Turkish (QSD-R-TR) | Problems with hypoglycemia |  |  |  |  | Worries about long-term complications |  | Physical complaints | Leisure time |  | Work  Relationship with partner  Doctor-patient relationship |  |  | Treatment regimen |
| Questionnaire on Stress in Patients with Diabetes-Revised-Turkish-Cyprus (QSD-R-TR) |  |  |  |  | Psycho-physiological aspects  Psychosocial distress  Outcomes | Psycho-physiological aspects  Outcomes | Psycho-physiological aspects  Fear/depression  Outcomes |  |  |  | Social  Medical relationships |  |  | Accountability |
| Sexual Quality of Life questionnaire-Female (SQOL-F) |  |  |  |  |  |  |  |  | Impact of sexual dysfunction on quality of life |  |  |  |  |  |
| Well-being questionnaire (W-BQ) 12 |  |  | Energy |  |  |  |  |  |  | Negative well-being  Positive well-being |  |  |  |  |
| Well-being questionnaire (W-BQ) 22 |  |  | Energy |  |  | Anxiety | Depression |  |  | Positive well-being |  |  | General well-being |  |
| Well-being questionnaire (W-BQ) 28 |  |  | Energy |  | Generic stress  Diabetes-specific stress |  |  |  |  | Generic negative well-being  Generic positive well-being  Diabetes-specific negative well-being  Diabetes-specific positive well-being |  |  |  |  |
| Well-being and Treatment Satisfaction scales (W-BQ) |  |  |  |  |  | Anxiety | Depression |  |  |  |  |  | Positive well-being |  |
| Well-being Enquiry for Diabetics (WED) | Symptoms |  |  |  |  |  |  |  |  | Discomfort |  | Impact |  | Serenity |
| The World Health Organisation- Five Well-Being Index (WHO-5) |  |  |  |  |  |  |  |  |  |  |  |  | Psychological well-being |  |
| World Health Organisation Quality of Life (WHOQOL-100) |  |  |  |  | Psychological | Psychological | Psychological | Physical |  |  | Social relationships |  |  | Environment  Level of independence |
| World Health Organisation Quality of Life (WHOQOL)-BREF |  |  |  |  | Psychological | Psychological | Psychological | Physical health |  |  | Social relationships |  |  | Environment |
